# Expression of HER2 Ultralow in Endometrial Carcinoma, High-Grade Serous Ovarian Cancer and Their Metastases

**DOI:** 10.3390/cancers18172843

**Published:** 2026-09-02

**Authors:** C. Backhaus, A. Gabriel, V. Guyon, D. Weiß, M. Werner, P. Bronsert, P. Groß, I. Juhasz-Böss, K. Kurowski

**Affiliations:** 1 Clinic for Gynecology and Obstetrics, University Medical Center of Freiburg, 79106 Freiburg, Germanydaniela.weiss@uniklinik-freiburg.de (D.W.); philipp.gross@uniklinik-freiburg.de (P.G.); 2 Institute of Pathology, University Medical Center of Freiburg, 79106 Freiburg, Germany; martin.werner@uniklinik-freiburg.de (M.W.); peter.bronsert@uniklinik-freiburg.de (P.B.); konrad.kurowski@uniklinik-freiburg.de (K.K.); 3 Core Facility for Histopathology and Digital Pathology, University Medical Center of Freiburg, 79106 Freiburg, Germany; 4 Comprehensive Cancer Center, University Medical Center of Freiburg, 79106 Freiburg, Germany

**Keywords:** endometrial carcinoma, high-grade serous ovarian carcinoma, HER2, HER2 ultra-low

## Abstract

**Background/Objectives:** HER2-directed antibody–drug conjugates have expanded the clinical relevance of low-level HER2 expression. However, the prevalence and clinical significance of HER2-ultralow expression in gynecologic malignancies remain insufficiently defined. This study characterized the full spectrum of HER2 expression in endometrial carcinoma (EC) and high-grade serous ovarian carcinoma (HGSOC), including matched metastatic lesions. **Methods:** HER2 expression was assessed by immunohistochemistry in tissue microarrays from 117 EC and 52 HGSOC patients. Available matched metastatic lesions were analyzed in 23 EC and 18 HGSOC cases. HER2 expression was categorized as zero, ultralow, 1+, 2+, or 3+. Associations with clinicopathological parameters and progression-free survival were evaluated. **Results:** HER2-ultralow expression was common in primary tumors, occurring in 49/117 EC cases (41.9%) and 11/52 HGSOC cases (21.2%), whereas HER2 3+ expression was rare (EC: 2/117, 1.7%; HGSOC: 1/52, 1.9%). HER2 expression in primary tumors was associated with progression-free survival in both entities, with HER2-zero tumors showing the longest estimated progression-free survival. HER2 score discordance between matched primary tumors and metastases occurred in 16/23 EC cases (69.6%) and 8/18 HGSOC cases (44.4%). **Conclusions:** HER2-ultralow expression represents a frequent and previously underrecognized category in EC and HGSOC. While its prognostic impact remains limited, the high prevalence of HER2-ultralow tumors suggests potential relevance for future HER2-targeted therapeutic strategies. Prospective studies are needed to determine whether this subgroup may benefit from a HER2-targeted therapy.

## Simple Summary

HER2-directed therapies are well established in breast cancer and have recently been extended to tumors with low and ultralow HER2 expression. In contrast, the role of HER2 in gynecological malignancies is still not well defined. In particular, little is known about tumors with very low HER2 expression, referred as HER2-ultralow. In this study, we analyzed HER2 expression in endometrial carcinoma and high-grade serous ovarian cancer, including matched metastatic lesions, to better characterize the full spectrum of HER2 expression. We found that HER2-ultralow tumors were common, accounting for 41.9% of primary endometrial carcinomas and 21.2% of primary high-grade serous ovarian carcinomas, whereas classical HER2- 3+ expression was rare. Recent clinical trials, such as DESTINY-PanTumor02, have demonstrated promising results of HER2-targeted therapies in gynecological cancers. Our findings suggest that more patients might potentially benefit from HER2-targeted treatment strategies in gynecological cancers and may help to guide future studies on targeted therapies in these patients.

## 1. Introduction

Endometrial cancer (EC) is one of the most frequently diagnosed cancers in women. 2025, more than 69,000 new cases were diagnosed in the United States while globally 420,368 new cases were estimated in 2022 [1,2]. The incidence of this disease has increased between 1990 and 2019 [3]. The majority of uterine corpus cancer are endometrial carcinoma, and the largest part of the endometrial carcinoma is taken up by endometrioid carcinoma [4]. Endometrial cancer is currently classified into four molecular subgroups based on its underlying molecular profile [5]. This classification enables more accurate prognostic stratification and supports a more individualized therapeutic approach [6].

In early-stage disease, treatment is primarily based on surgery, consisting of total hysterectomy and bilateral salpingo-oophorectomy, with lymphadenectomy or sentinel lymph node dissection performed when indicated [6]. However, in advanced or metastatic disease, immune checkpoint inhibitors have become increasingly important. Mismatch-repair-(MMR)-proficient patients are now treated with Pembrolizumab according to the KEYNOTE-158 study [7] and patients with an MMR-deficient tumor with Dostarlimab according to the RUBY study [8]. To date, the efficacy of antibody drug conjucates (ADCs) are investigated within endometrial cancer patients (Mirvetuximab-Soravtansine, Trastuzumab-Deruxtecan (T-DXd), DB-1303, Sacituzumab-Govitecan) [9].

Ovarian cancer (OC) remains the most lethal gynecologic malignancy in women. In the United States, an estimated 20,890 new cases were expected in 2025, while 324,603 new cases were reported worldwide in 2022 [10]. Most patients are diagnosed with advanced-stage disease, largely because symptoms are absent or nonspecific in the early phases of the disease [11]. In the United States, the overall 5-year relative survival rate is 43%. Histologically, high-grade serous ovarian carcinoma (HGSOC) represents the predominant subtype [12].

The cornerstone of treatment for ovarian carcinoma remains cytoreductive surgery followed by platinum-based chemotherapy [13]. However, given the high rate of recurrence, antibody–drug conjugates (ADCs) are gaining increasing clinical relevance [9]. Mirvetuximab-Soravtansine, for example, showed promising results for patients with platinum-resistant ovarian cancer [14]. Further ADCs are also investigated currently [9].

The human epidermal growth factor receptor 2 (also known as HER2, ErbB22 or p 1985) is a 185 kDa Glycoprotein with tyrosine kinase activity located on the long arm of human chromosome 17 (17q12) [15]. Its extracellular ligand domain is 632 amino acids long. It plays a crucial role in regulating cell growth and differentiation [16]. Across a range of malignancies, HER2 overexpression is associated with more aggressive tumor behavior and poorer clinical outcome [16].

Around 15–20% of breast cancers show HER2 positivity [17], and HER2-targeted therapies have become a gold standard in the treatment for these patients [18].

HER2-positive breast cancer is initially identified by immunohistochemical assessment of HER2 expression. Tumors are scored on a scale from 0 to 3+, with scores of 0 and 1+ considered HER2-negative, and a score of 3+ considered HER2-positive. In tumors with an equivocal IHC score of 2+, additional in situ hybridization (ISH) is performed to clarify HER2 status [19].

In breast cancer, the traditional binary distinction between HER2-positive and HER2-negative disease is increasingly being replaced by a more differentiated classification into HER2-positive, HER2-low and HER2-zero. HER2-positive tumors are defined as IHC 3+ or IHC 2+ with positive ISH, whereas HER2-low tumors show IHC 1+ or IHC 2+ with negative ISH, and HER2-zero tumors are classified as IHC 0 [17]. Those HER2-low tumors already get treated with anti-HER2 therapies. Notably, the Destiny-BREAST04 trial yielded promising results in patients with HER2-low breast cancer [20]. Nevertheless, the HER2-ultralow status is currently gaining prominence. It refers to tumors that do not meet the criteria for HER2 score 1+ but still show some membranous HER2 expression in tumor cells. HER2-ultralow is not a separate positive HER2 score but an operational subdivision within conventional IHC score 0. It corresponds to IHC 0 with membrane staining, also referred to as 0+, and is defined by faint or barely perceptible incomplete membranous staining in >0% but ≤10% of tumor cells. In contrast, true HER2-zero tumors show no membranous staining, whereas HER2 1+ requires faint incomplete membranous staining in >10% of tumor cells [19].

This distinction is clinically relevant because very low HER2 expression may still be sufficient for binding of HER2-directed ADCs. More recently, the DESTINY-Breast06 trial evaluated the efficacy of T-DXd, a HER2-directed ADC, in patients with HER2-ultralow breast cancer. The trial demonstrated that approximatively 20–25% of hormone receptor-positive/HER2 negative breast cancers can be classified as HER2-ultralow. Preliminary results from this trial have shown promising clinical outcomes [21,22,23].

Both endometrial and ovarian cancer can express HER2 [24].

HER2 expression is not restricted to neoplastic tissue, as low-to-moderate immunoreactivity has been reported in normal endometrial glandular epithelium [25] and weak staining in normal ovarian surface epithelium [26]. Thus, low-level HER2 expression may also represent a physiological feature of non-neoplastic gynecologic epithelium. HER2 expression plays a significant role in EC. HER2 testing is recommended for serous EC, as it may have therapeutic implications [27]. While HER2 overexpression has been observed in uterine endometrioid carcinoma, its prevalence is notably lower compared to serous carcinoma. HER2 overexpression of uterine serous carcinoma has been reported in 14–80% [24].

The data on HER2 expression in serous ovarian carcinoma (OC) are inconsistent [28]. Some studies report low rates of HER2 positivity (6.6%), while others suggest slightly higher rates. Overall, HER2 overexpression or amplification is less common in HGSOC compared to mucinous subtypes (approximatively 10–20%) [28]. In OC patients, HER2 overexpression has been associated with resistance to chemotherapy and shortened survival [29,30].

There are no data on HER2-ultralow in EC and OC so far. Thus, HER2-targeted therapies are emerging as a potential option for ovarian and endometrial cancer patients. In the DESTINY-PanTumor02 trial, T-DXd achieved an overall response rate (ORR) of 45.0% (95% CI, 29.3–61.5) in patients with ovarian cancer, with even higher response rates observed in tumors showing strong HER2 expression (IHC 3+; 63.6%). In contrast, trastuzumab monotherapy has thus far shown only limited clinical impact in ovarian cancer, with a reported ORR of 7.3% [31]. In patients with endometrial cancer, the DESTINY-PanTumor02 trial reported an overall response rate (ORR) of 57.5% (95% CI, 40.9–70.3), with better responses in tumors with high HER2 expression, where the ORR reached 85.0% in the IHC 3+ subgroup [31].

These data indicate that T-DXd has the potential to become a highly relevant treatment option for patients with these challenging tumor entities. However, this study primarily addressed HER2-expressing tumors and does not directly define the therapeutic relevance of HER2-ultralow disease. Thus, whether very low HER2 expression may represent a clinically meaningful target in EC or HGSOC remains unresolved.

Therefore, the aim of this study was to characterize the full spectrum of HER2 expression in EC and HGSOC, focusing on the HER2-ultralow subgroup. We assessed HER2 expression in primary tumors and available matched metastatic lesions, evaluated associations with clinicopathological parameters and progression-free survival, and analyzed HER2 score concordance between primary and metastatic tissue. By specifically including the HER2-ultralow category, this study aimed to determine whether very low-level HER2 expression represents a relevant and previously underrecognized subgroup in these gynecologic malignancies.

These data may provide a rationale for future studies exploring whether HER2-directed therapies could be expanded beyond currently established indications to include a broader population of patients with EC or HGSOC.

## 2. Materials and Methods

This study was approved by the Ethics Committee of the Albert-Ludwig-University of Freiburg, Germany (reference number 437/20 and 20-1263). Written and informed consent was obtained from all patients alive at the time of inclusion.

### 2.1. Human Tissue Samples

In this study, 117 patients with histologically confirmed EC and 52 patients with histologically confirmed HGSOC were included.

No patient received neoadjuvant therapy, and all patients were primarily surgically resected at the Department for Gynecology and Obstetrics of the University Medical Hospital, Freiburg, Germany.

Tumor stage was defined in accordance with the International Federation of Gynecology and Obstetrics (FIGO) [32,33]; tumor grade and stadium were determined according to the World Health Organization criteria (WHO) [34]. All cases were reviewed by a board-certified pathologist prior to inclusion.

Patients with a history of another malignancy, prior primary systemic therapy, or no written informed consent were excluded. Following application of these exclusion criteria, the final study cohort comprised 117 patients. The samples were collected from the Institute for Clinical Pathology (ICP) of the University Hospital in Freiburg, Germany.

### 2.2. Tissue Microarray Construction

Tissue microarrays (TMAs) were constructed as previously described [35]. Briefly, digitized and pseudonymized hematoxylin and eosin stained slides (PANNORAMIC^®^ 1000-Scanner, 3DHISTECH Ltd., Budapest, Hungary) were annotated with circular tumor regions of interest (ROIs) by a board-certified pathologist using the Caseviewer software (Sysmex, v. 2.3, Kobe, Japan) [35]. For each patient, four ROIs from the primary tumor—two from the tumor center, two from the tumor periphery—were assigned. Furthermore, up to four ROIs of metastatic tumor samples were annotated, if available.

Metastatic tissue samples comprised both surgical resection and biopsy specimens. TMAs were then constructed using the TMA Grand Master (3DHISTECH Ltd., Budapest, Hungary). Additionally, three cores each of de-identified archival human placental and tonsillar tissue as additional non-neoplastic on-slide reference tissues. These tissues were not used as the primary HER2-positive control, as quantitative staining standards, or to determine the validity of an HER2 staining run. Following completion of the tissue-core transfer, the TMA block underwent six heating–cooling cycles according to a previously published protocol [35]. In each cycle, the block was incubated at 37 °C for 60 min and then immediately cooled on crushed ice for 10 min. This procedure was performed to improve the integration and adhesion of the donor tissue cores within the surrounding paraffin of the recipient block, thereby reducing gaps, core displacement, and core loss during subsequent sectioning.

### 2.3. Immunohistochemistry

HER2 immunohistochemistry was performed on 2 µm formalin-fixed, paraffin-embedded TMA sections using the prediluted PATHWAY anti-HER-2/neu (4B5) rabbit monoclonal primary antibody (Ventana Medical Systems, Inc./Roche Diagnostics, Tucson, AZ, USA) on a Ventana BenchMark ULTRA automated staining instrument (Roche Diagnostics, Tucson, AZ, USA). Following automated deparaffinization and antigen retrieval, antibody binding was visualized using the Ventana detection kit OptiView (Roche Diagnostics, Tucson, AZ, USA), and the sections were counterstained with hematoxylin in accordance with the manufacturer’s instructions [36]. 

The staining protocol had been validated for routine diagnostic use. Each staining run included a previously validated formalin-fixed, paraffin-embedded human HER2-positive and negative breast-carcinoma control. A staining run was accepted only when the control demonstrated the expected membranous staining pattern and intensity.

The analytical performance of the 4B5 antibody on the Ventana BenchMark platform has previously been evaluated against alternative immunohistochemical assays and HER2 in situ hybridization [37,38]. The assay was applied to EC and HGSOC tissue for research purposes, and HER2 staining was interpreted analogously to the ASCO/CAP breast cancer scoring system as described below.

### 2.4. HER2 Expression Evaluation

Immunohistochemically stained TMA slides were digitized using a PANNORAMIC^®^ 1000 scanner (3DHISTECH Ltd., Budapest, Hungary) and evaluated with QuPath image analysis software (v0.30) [39] by two board-certified pathologists.

HER2 immunohistochemical staining was evaluated analogously to the ASCO/CAP breast cancer scoring algorithm, taking into account membranous staining intensity, completeness, and the percentage of stained invasive tumor cells [19]. HER2 score 0 was assigned when no membranous staining was observed or when faint or barely perceptible, incomplete membranous staining was present in ≤10% of tumor cells. For the purposes of the present study, score 0 tumors were further subdivided into HER2-zero, defined as the complete absence of membranous staining, and HER2-ultralow, defined as faint or barely perceptible, incomplete membranous staining in >0% but ≤10% of tumor cells. HER2 score 1+ was defined as faint or barely perceptible, incomplete membranous staining in >10% of tumor cells. HER2 score 2+ was defined as weak to moderate complete membranous staining in >10% of tumor cells or intense complete membranous staining in ≤10% of tumor cells. HER2 score 3+ was defined as intense, complete circumferential membranous staining in >10% of tumor cells [19]. HER2-ultralow was therefore treated as an operational subclassification within the conventional IHC 0 category, consistent with the definition of IHC 0 with membranous staining used in DESTINY-Breast06 [40].

Representative histological images of HER2 immunohistochemistry and the control tissue were captured from digitally scanned slides using HALO AI image analysis software (Indica Labs, Albuquerque, NM, USA) and are provided in the Supplementary Materials (Supplementary Figures S1A–J and S2).

### 2.5. Statistical Analysis

Statistical analysis was performed in R (version 4.2.2; R Foundation for Statistical Computing, Vienna, Austria) using RStudio (desktop version 2022.07.2+576; RStudio, PBC, Boston, MA, USA). All analyses were carried out with a custom-coded script, as previously described [35] or survival analyses, the survival (v. 3.2-13), gtsummary (v. 2.0.1), and cutpointr (v. 1.1.2) packages were applied. To evaluate progressive free survival (PFS), patients were stratified according to their HER2-Score. 

Survival probabilities were estimated using the Kaplan–Meier method, and differences between groups were assessed with the log-rank test. 

For multivariable analyses, Cox proportional hazards regression models were fitted. Variables significantly associated with progression-free survival in univariable analyses were considered for inclusion in the multivariable model. To avoid multicollinearity, FIGO stage and the individual TNM variables were not included simultaneously in the multivariable model, as the FIGO classification already incorporates tumor extent, nodal status, and distant metastasis. 

Cox regression analyses were supplemented by Firth’s penalized-likelihood Cox regression as a sensitivity analysis using the coxphf package (version 1.13.4) [41]. The original five HER2 categories—HER2-zero, HER2-ultralow, HER2 1+, HER2 2+, and HER2 3+—were retained, with HER2-zero serving as the reference category. The multivariable penalized models contained the same covariates as the corresponding conventional Cox models. The overall association between the four non-reference HER2 categories and progression-free survival was assessed using a four-degree-of-freedom penalized likelihood-ratio test. The conventional Cox models were considered the primary analyses, whereas the Firth models were considered sensitivity analyses.

For bivariate analyses, Fisher’s exact test was used to examine associations between categorical variables, whereas the Kruskal–Wallis rank-sum test was applied for comparisons involving continuous or ordinal variables across groups.

A two-sided *p*-value < 0.05 was considered statistically significant.

## 3. Results

### 3.1. Patient Characteristics and Clinicopathological Data

A total of 117 patients with EC were included. The mean age at diagnosis was 67 years (median 67, range 41–91 years). Histologically, the majority of tumors were endometrioid carcinomas (*n* = 112, 95.7%), followed by serous carcinomas (*n* = 3, 2.6%), clear cell carcinoma (*n* = 1, 0.9%), and mixed serous/endometrioid carcinoma (*n* = 1, 0.9%). Due to the small numbers of non-endometrioid subtypes, these tumors were grouped together and analyzed as “other” in subsequent analyses. Most tumors were diagnosed at an early stage, with 75 patients (64%) classified as FIGO stage I, 9 patients (7.7%) as stage II, 23 patients (20%) as stage III, and 10 patients (8.5%) as stage IV.

Pathological tumor staging showed pT1 disease in 83 patients (71%), pT2 in 13 patients (11%), pT3 in 20 patients (17%), and pT4 in 1 patient (0.9%). Regarding nodal status, 37 patients (32%) were pN0, 21 patients (18%) pN1, while 59 patients (50%) had unknown nodal status (pNx). Distant metastases were present in 24 patients (21%), whereas 93 patients (79%) were classified as pM0.

Lymphatic vessel invasion (L1) was observed in 30 patients (26%), while 87 patients (74%) showed no lymphatic invasion (L0). Venous invasion (V1) was detected in 9 patients (7.7%), whereas 108 patients (92%) were V0. Perineural invasion (Pn1) was not observed in this cohort (0%). Resection margins were negative (R0) in 103 patients (88%), microscopically positive (R1) in 9 patients (7.7%), and 5 cases (4.3%) were classified as Rx.

The mean PFS was 68 months (median 57 months, range 1–206 months), and disease progression occurred in 59 patients (50%).

The OC cohort comprised 52 patients. The mean age at diagnosis was 64 years (median 65, range 44–87 years). Based on FIGO stage, the majority of tumors presented at an advanced stage: 4 patients (7.7%) had FIGO stage I disease, 7 patients (13%) had stage II, 25 patients (48%) had stage III, and 16 patients (31%) had stage IV disease.

With respect to pathological tumor stage, 4 patients (7.7%) had pT1 tumors, 8 patients (15%) had pT2 tumors, and 40 patients (77%) had pT3 disease. For nodal involvement, 18 patients (35%) were classified as pN0, 23 patients (44%) as pN1, and 11 patients (21%) had not received lymphadenectomy (pNx). Concerning distant spread, 36 patients (69%) were staged as pM0 and 16 patients (31%) as pM1.

Lymphatic vessel invasion (L1) was identified in 24 patients (46%), whereas 28 patients (54%) had no evidence of lymphatic invasion (L0). Venous invasion (V1) was identified in 4 patients (7.7%), while 48 patients (92%) were categorized as V0. Perineural invasion (Pn1) was documented in 1 patient (1.9%), whereas 51 patients (98%) were Pn0. Residual tumor status was recorded as R0 in 43 patients (83%), R1 in 7 patients (13%), and Rx in 2 patients (3.8%).

The mean progression-free survival was 28 months (median 16 months, range 1–129 months), with 49 patients (94%) experiencing disease progression.

The clinicopathological data of OC and EC are also summarized in Table 1.

### 3.2. Immunohistochemical HER2 Expression

HER2 immunohistochemistry in primary endometrial carcinoma tumors revealed HER2-Score 0 in 58 cases (50%), ultralow expression in 49 cases (42%), HER2-Score 1+ in 7 cases (6.0%), HER2-Score 2+ in 1 case (0.9%), and HER2-Score 3+ in 2 cases (1.7%). Among metastatic lesions, HER2-Score 0 was observed in 12 cases (52%), HER2-ultralow in 7 cases (30%), 1+ in 3 cases (13%), 2+ in 1 case (4.3%), and no cases showed HER2-Score 3+ expression (Table 1). Metastatic sites analyzed included the liver, lung, non-pelvic peritoneum, non-regional lymph nodes, and spleen. 

Age at diagnosis according to primary-tumor HER2 expression was HER2 Zero: mean 66.0 years (median 65.0, range 43.0–91.0; *n* = 58); Ultralow: mean 67.6 years (median 68.0, range 41.0–88.0; *n* = 49); HER2 Score 1+: mean 70.6 years (median 66.0, range 63.0–87.0; *n* = 7); HER2 Score 2+: mean 64.0 years (median 64.0, range 64.0–64.0; *n* = 1); HER2 Score 3+: mean 65.0 years (median 65.0, range 64.0–66.0; *n* = 2). Age did not differ significantly across HER2 categories (Kruskal–Wallis *p* = 0.822) (Supplementary Table S1).

Evaluation of HER2 immunohistochemistry in primary ovarian tumors showed HER2-Score 0 in 38 cases (73%) and HER2-ultralow expression in 11 cases (21%); HER2-Scores 1+, 2+, and 3+ were each found in 1 case each (1.9%). In the metastatic lesions, HER2-Score 0 was present in 13 cases (72.2%), ultralow expression in 4 cases (22.2%), and HER2-Score 2+ in 1 case (5.6%); no mestastais demonstrated HER2-Score 1+ or 3+ staining (Table 1). Metastatic sites analyzed included the liver, sigmoid, lymph nodes, omentum, and gallbladder. 

Age at diagnosis according to primary-tumor HER2 expression was HER2-zero: mean 63.2 years (median 63.6, range 44.0–86.5; *n* = 38); ultralow: mean 69.7 years (median 69.6, range 57.6–84.3; *n* = 11); HER2 Score 1+: mean 49.0 years (median 49.0, range 49.0–49.0; *n* = 1); HER2 Score 2+: mean 70.8 years (median 70.8, range 70.8–70.8; *n* = 1); HER2 Score 3+: mean 51.9 years (median 51.9, range 51.9–51.9; *n* = 1). Age did not differ significantly across HER2 categories (Kruskal–Wallis *p* = 0.175) (Supplementary Table S2).

### 3.3. Logrank-Test of Progressive-Free Survival

**Figure 1:**
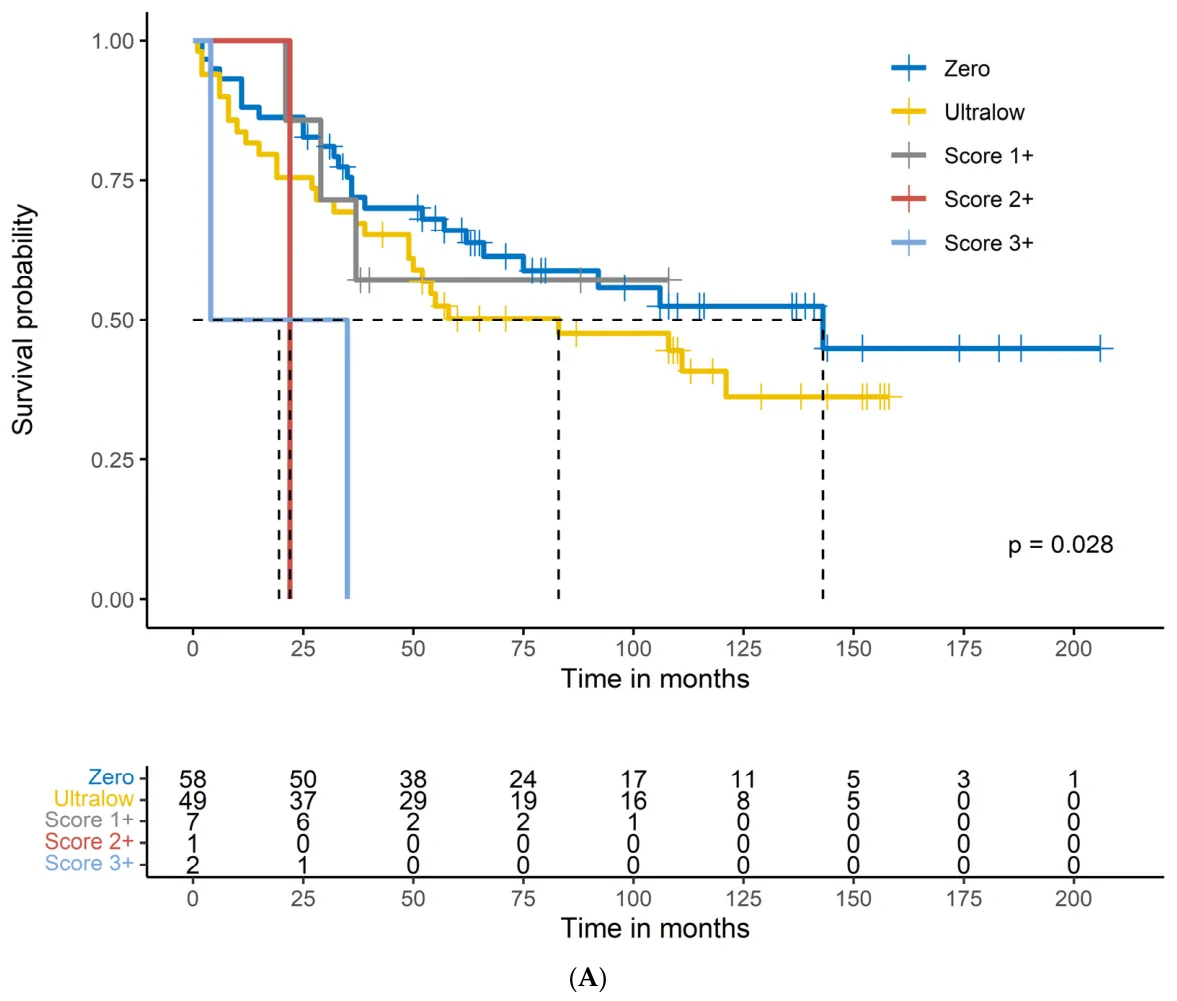

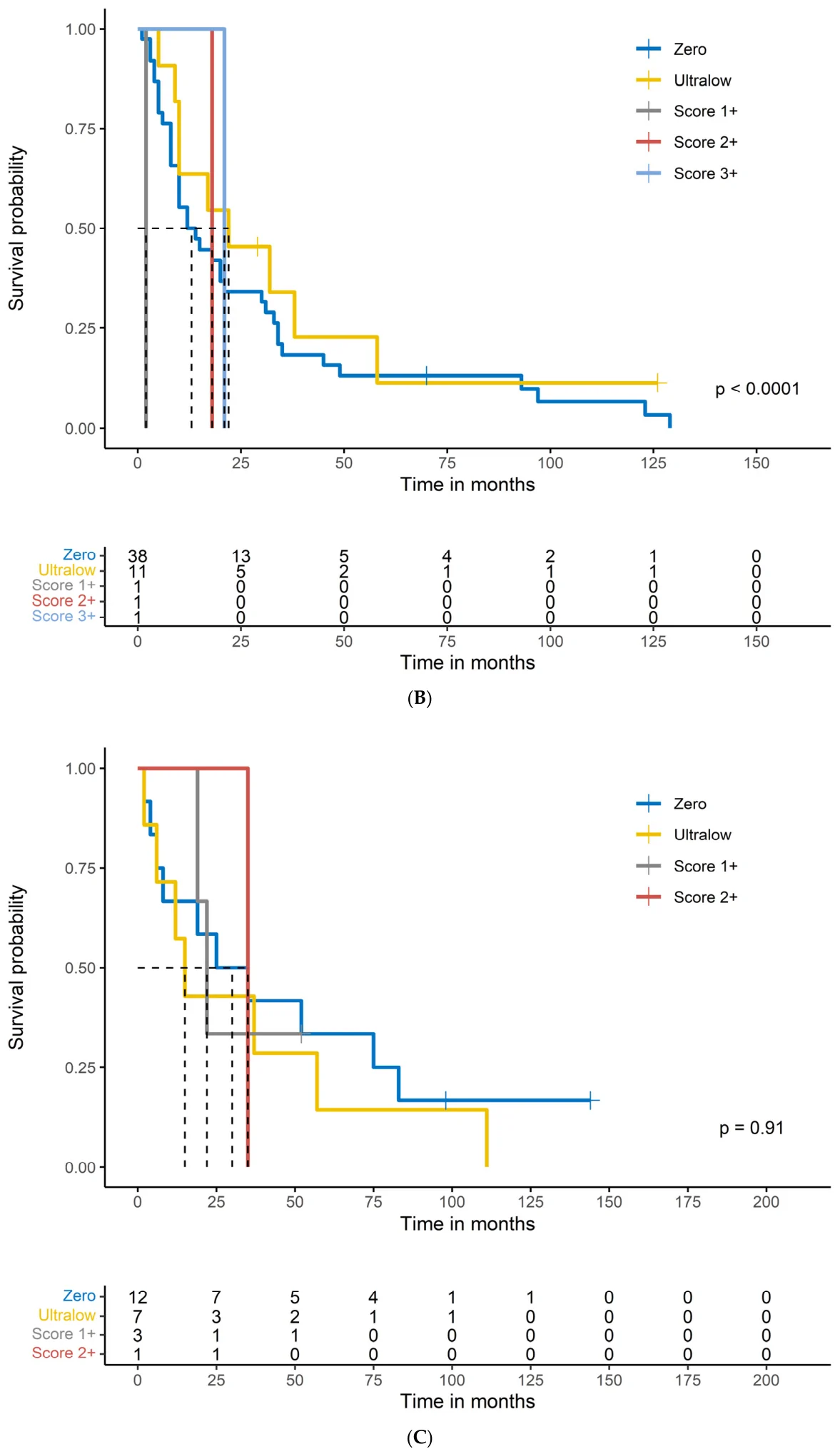

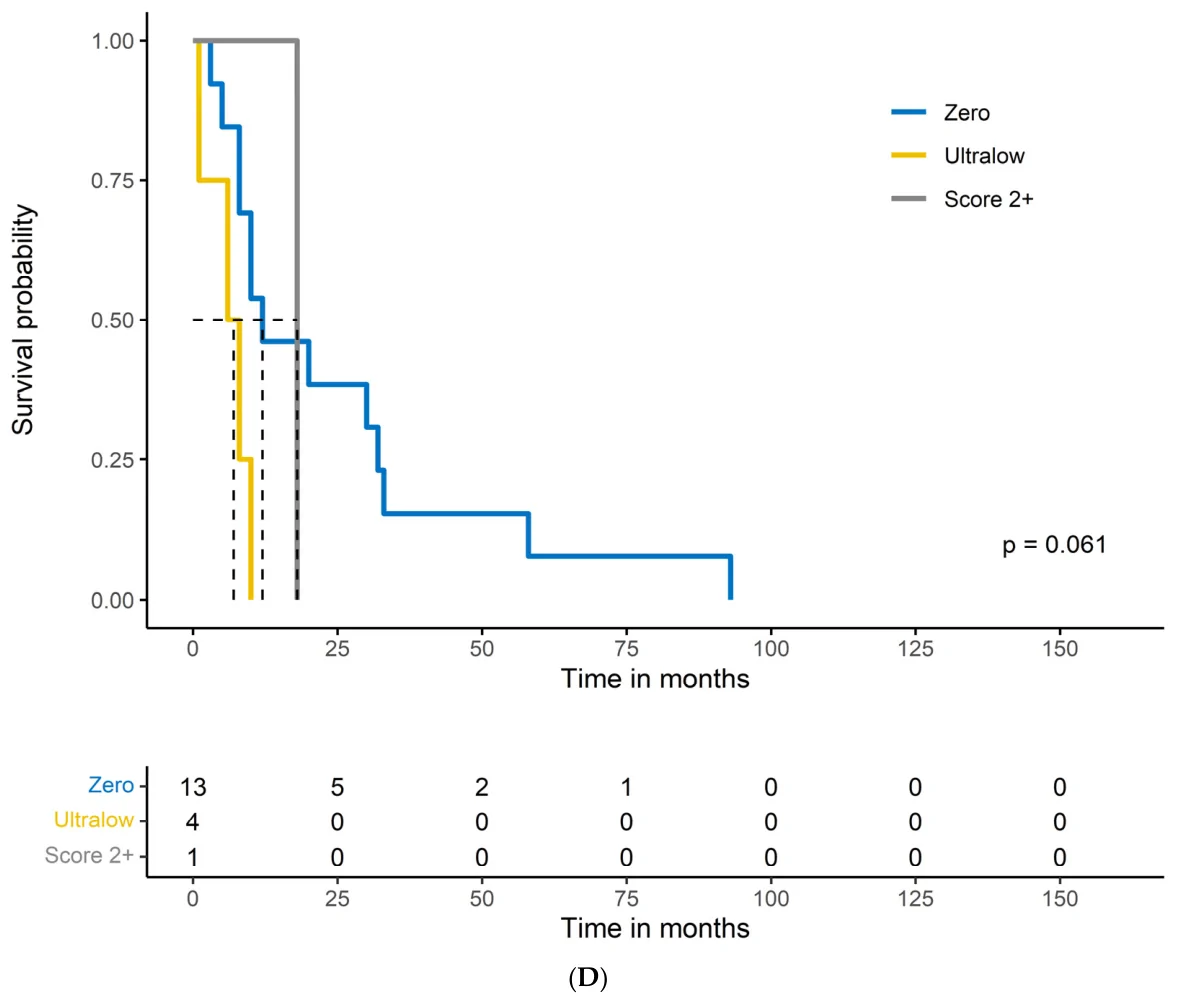
(**A**) Kaplan–Meier analysis of progression-free survival according to HER2 expression in primary endometrial carcinoma. (**B**) Kaplan–Meier analysis of progression-free survival according to HER2 expression in primary HGSOC. (**C**) Kaplan–Meier analysis of progression-free survival according to HER2 expression in metastatic lesions of endometrial carcinoma. (**D**) Kaplan–Meier analysis of progression-free survival according to HER2 expression in metastatic lesions of HGSOC. The dashed lines indicate the median survival for each group, defined as the time point at which the estimated survival probability reaches 50%.

In EC and OC, HER2 expression showed a significant impact on PFS (respectively *p* = 0.028 and *p* < 0.0001), with the HER2-zero subpopulation showing the longest estimated PFS (Figure 1A,B).

The HER2 expression in metastasis did not demonstrate significant differences in PFS (respectively *p* = 0.91 and *p* = 0.061) (Figure 1C,D).

Progression-free survival was compared according to HER2 expression categories in primary EC tumors (Figure 1A), primary HGSOC tumors (Figure 1B), metastatic EC lesions (Figure 1C), and metastatic HGSOC lesions (Figure 1D).

### 3.4. Cox Regression of Progressive-Free Survival

In EC, HER2 expression showed no consistent significant association across most expression levels (Table 2). Compared to HER2-negative tumors, ultralow expression (Hazard Ratio (HR): 1.39; 95% Confidence Interval (CI): [0.81, 2.39], *p* = 0.2) and HER2 1+ (HR: 1.21; 95% CI: [0.36, 4.02], *p* = 0.8) were not significantly associated with PFS. HER2 2+ demonstrated a trend toward worse PFS (HR: 5.89; 95% CI: [0.78, 44.8], *p* = 0.087), while HER2 3+ was significantly associated with poorer PFS (HR: 6.44; 95% CI: [1.49, 27.8], *p* = 0.013).

In the multivariable analysis, HER2 expression lost its prognostic significance. Neither ultralow HER2 expression (HR: 1.01; 95% CI: 0.56–1.81; *p* > 0.9), HER2 1+ expression (HR: 0.75; 95% CI: 0.17–3.29; *p* = 0.7), HER2 2+ expression (HR: 1.90; 95% CI: 0.20–17.7; *p* = 0.6), nor HER2 3+ expression (HR: 1.91; 95% CI: 0.30–12.3; *p* = 0.5) showed a statistically significant association with progression-free survival in multivariable Cox regression analysis. Among clinicopathological covariables, age remained independently associated with progression-free survival in multivariable Cox regression analysis (univariable: HR: 1.05; 95% CI: 1.03–1.08; *p* < 0.001; multivariable: HR: 1.08; 95% CI: 1.05–1.12; *p* < 0.001). In addition, pT2 stage (multivariable: HR: 3.68; 95% CI: 1.38–9.82; *p* = 0.009) and distant metastasis (pM1 multivariable: HR: 4.33; 95% CI: 1.78–10.5; *p* = 0.001) were independently associated with progression-free survival. Stage pT3 showed a borderline association in the multivariable model (HR: 2.53; 95% CI: 1.00–6.43; *p* = 0.051), whereas lymph node involvement, lymphovascular invasion, and residual tumor status did not remain statistically significant.

Results of the Cox regression analysis are shown in Table 2.

Firth’s penalized-likelihood Cox regression yielded findings consistent with the conventional Cox analysis. The global HER2 effect was significant in the univariable penalized model (*p* = 0.047). HER2 3+ expression was associated with poorer PFS compared with HER2-zero expression (HR 7.67; 95% profile penalized-likelihood CI 1.53–24.20; (*p* = 0.018)), while HER2 2+ expression showed a borderline association (HR 8.59; 95% CI 0.94–34.62; (*p* = 0.055)). HER2-ultralow and HER2 1+ expression were not significantly associated with PFS. After adjustment, none of the individual HER2 categories remained statistically significant. Because the HER2 2+ and HER2 3+ groups comprised only one and two patients, respectively, these category-specific findings were considered exploratory. Complete penalized regression results are provided in Supplementary Table S3.

In HGSOC, HER2 expression showed heterogeneous associations with PFS (Table 3). Compared with HER2-negative tumors, ultralow HER2 expression was not significantly associated with PFS in univariable analysis (HR: 0.71; 95% CI: 0.34–1.47; *p* = 0.4). In contrast, HER2 1+ expression was significantly associated with worse PFS (HR: 47.5; 95% CI: 2.96–762; *p* = 0.006), whereas HER2 2+ expression (HR: 1.22; 95% CI: 0.16–9.08; *p* = 0.8) and HER2 3+ expression (HR: 0.98; 95% CI: 0.13–7.28; *p* > 0.9) showed no significant association.

In multivariable analysis, HER2 1+ expression remained independently associated with worse PFS (HR: 34.8; 95% CI: 1.95–619; *p* = 0.016). Ultralow HER2 expression (HR: 0.94; 95% CI: 0.44–2.03; *p* = 0.9), HER2 2+ expression (HR: 1.5; 95% CI: 0.2–11.5; *p* = 0.7), and HER2 3+ expression (HR: 1.15; 95% CI: 0.15–8.75; *p* = 0.9) were not significantly associated with PFS after adjustment.

Among the clinicopathological covariates, pT3 disease (HR 4.94; 95% CI 1.07–22.7; *p* = 0.040), venous invasion (HR 3.63; 95% CI 1.17–11.3; *p* = 0.025), and Rx status (HR 9.10; 95% CI 1.77–46.7; *p* = 0.008) were associated with poorer PFS. Results of the Cox regression analysis are shown in Table 3.

Firth’s penalized-likelihood Cox regression yielded category-specific estimates similar to those of the conventional Cox analysis. However, the global HER2 effect did not reach statistical significance in either the univariable model (*p* = 0.059) or the adjusted model (*p* = 0.126). The single HER2 1+ case generated a high hazard ratio in the univariable model (HR 47.86; 95% profile penalized-likelihood CI 3.87–591.34; (*p* = 0.006)) and after adjustment (HR 33.06; 95% CI 2.47–448.37; (*p* = 0.012)). HER2-ultralow, HER2 2+, and HER2 3+ expression were not significantly associated with PFS. Because each of the HER2 1+, 2+, and 3+ categories contained only one patient, these results were interpreted as exploratory. Complete penalized regression results are provided in Supplementary Table S4.

### 3.5. Bivariate Analysis

Bivariate analysis in EC revealed significant associations between metastatic HER2 expression and FIGO stage (*p* = 0.024), as well as between primary HER2 expression and pM status (*p* = 0.006). No further significant correlations were observed.

Bivariate analysis in HGSOC showed no significant associations between HER2 expression in primary tumors and PFS or most clinicopathological variables. The only significant correlation in the primary setting was found between HER2 expression in primary tumors and matched metastases (*p* = 0.030). In metastatic lesions, HER2 expression was significantly associated with resection status (*p* = 0.012), while no further significant associations were detected.

Detailed bivariate analysis results are shown in the Supplementary Tables S5 and S6.

### 3.6. HER2 Expression in Matched Primary Tumors and Metastatic Lesions

Matched primary tumor and metastatic tissue was available for 23 patients with EC and 18 patients with HGSOC. In EC, HER2 expression differed between primary tumors and matched metastatic lesions in 16 of 23 cases (69.6%) (Table 4), whereas 7 cases (30.4%) showed an unchanged HER2 score. A decrease in HER2 expression in the metastatic lesion was observed in 11 cases (47.8%), while 5 cases (21.7%) showed a higher score in the metastatic lesion.

The most frequent transition was from HER2-ultralow in the primary tumor to HER2-zero in the metastasis (seven cases, 30.4%). Conversely, three HER2-zero primary tumors shifted to HER2-ultralow in the metastasis (13.0%), and two HER2-ultralow primary tumors shifted to HER2 1+ in the metastasis (8.7%). HER2 3+ expression was not retained in metastatic lesions: one HER2 3+ primary tumor shifted to HER2-zero, and one shifted to HER2 1+.

Exact agreement between primary and metastatic tissue was 30.4% with a weighted kappa of κ = 0.338 (*p* = 0.086). The Wilcoxon signed-rank test showed a non-significant trend toward lower HER2 expression in metastatic tissue (*p* = 0.073).

In HGSOC, HER2 expression differed between primary tumors and matched metastatic lesions in 8 of 18 cases (44.4%) (Table 4), whereas 10 cases (55.6%) showed an unchanged HER2 score.

The most frequent pattern was stable HER2-zero expression in both the primary tumor and metastatic tissue (nine cases, 50.0%). Four HER2-zero primary tumors shifted to HER2-ultralow in the metastasis (22.2%), whereas four HER2-ultralow primary tumors shifted to HER2-zero in the metastasis (22.2%). One case retained HER2 2+ expression in both the primary tumor and metastatic tissue (5.6%). No transitions involving HER2 1+ or HER2 3+ expression were observed.

Exact agreement between primary and metastatic tissue was 55.6% with a weighted kappa of κ = 0.333 (*p* = 0.157). The Wilcoxon signed-rank test showed no significant difference in HER2 expression between primary and metastatic tissue (*p* = 1.0).

The complete HER2 transition matrices for EC and HGSOC, illustrating the exact score shifts between matched primary tumors and metastatic lesions, are provided in Supplementary Tables S7 and S8. Representative paired HER2 immunohistochemical images illustrating both decreased and increased HER2 expression in metastatic lesions compared with the corresponding primary tumors are provided in Supplementary Figures S3–S6 (EC: Figures S3 and S4; HGSOC: Figures S5 and S6).

## 4. Discussion

In this study, we characterized the spectrum of HER2 expression in primary EC and HGSOC, including matched metastatic lesions, with a particular focus on the HER2-ultralow subgroup. The central finding is that HER2-ultralow expression represents a substantial proportion of cases in both tumor entities, whereas classical HER2-positive tumors are rare. In our cohorts, HER2-ultralow expression was observed in 42% of EC and in 21% of HGSOC, while HER2 IHC 2+/3+ expression was present in only 2.6% and 3.8%, respectively. These data suggest that a purely binary distinction between HER2-positive and HER2-negative disease is insufficient in these gynecologic malignancies and that a relevant subset of patients may fall into a biologically and potentially therapeutically meaningful intermediate category.

From a clinical perspective, our findings are noteworthy because HER2-directed ADCs have shifted the therapeutic landscape beyond the classical HER2-positive setting. From a clinical perspective, our findings are relevant in light of the DESTINY-Breast06 trial, which extended the therapeutic relevance of HER2 expression to the HER2-low and HER2-ultralow spectrum in hormone receptor-positive metastatic breast cancer. In this phase III study, T-DXd improved PFS compared with standard chemotherapy in patients with HER2-low disease after progression on endocrine therapy, and clinically meaningful benefit was also observed in the HER2-ultralow subgroup. Based on these data, the Food and Drug Administration (FDA) approved T-DXd for unresectable or metastatic HR-positive HER2-low or HER2-ultralow breast cancer after progression on at least one endocrine therapy [40]. Although these results cannot be directly extrapolated to gynecologic malignancies, they support the biological and clinical rationale for evaluating HER2-ultralow expression as a potential therapeutic biomarker in EC and HGSOC.

Nevertheless, our data support the hypothesis that HER2-directed therapy could become relevant even for patients with HER2-ultralow gynecologic tumors, particularly because HER2-ultralow cases clearly outnumber HER2-positive cases in our cohorts. If HER2-ultralow expression proves predictive for ADC response, the population potentially eligible for treatment could exceed the number of conventionally HER2-positive patients. This concept is biologically plausible, as the antitumor effect of T-DXd is thought to rely not only on target binding and internalization but also on payload release with a bystander effect, a mechanism that may be particularly relevant in tumors with low or heterogeneous HER2 expression [42].

Extrapolation from breast cancer to endometrial and ovarian cancer is not straightforward. A major issue is that HER2 assessment is tumor-type dependent. In breast cancer, HER2 scoring was developed for tumors that typically show complete circumferential membranous staining when amplified [43]. In contrast, uterine serous carcinoma frequently exhibits basolateral or lateral membranous staining and marked intratumoral heterogeneity, features that resemble gastric or gastroesophageal adenocarcinoma more than breast cancer [27]. Reviews of HER2 testing in uterine serous carcinoma emphasize that these staining patterns complicate the direct transfer of breast-derived scoring concepts to gynecologic cancers [27].

When comparing HER2 scoring systems, the most relevant differences between breast cancer and gastric/gastroesophageal adenocarcinoma scoring concern the interpretation of HER2-positive tumors. According to breast cancer-based scoring, HER2 3+ requires intense, complete circumferential membranous staining in >10% of tumor cells, whereas HER2 2+ is defined by weak to moderate complete membranous staining in >10% of tumor cells, or intense complete membranous staining in ≤10% of tumor cells [43]. In contrast, gastric cancer scoring allows strong complete, basolateral, or lateral membranous staining in ≥10% of tumor cells for HER2 3+ in surgical specimens, while HER2 2+ is defined by weak to moderate complete, basolateral, or lateral membranous reactivity in ≥10% of tumor cells. In gastric biopsy specimens, the percentage threshold is not applied in the same way; instead, a cluster of at least five tumor cells with the respective staining pattern may be sufficient, irrespective of the overall percentage of positive tumor cells [44].

These differences could theoretically influence HER2 classification in gynecologic tumors, particularly in cases with basolateral or lateral staining patterns. Accordingly, some tumors might have been classified differently in our study if gastric rather than breast cancer-based criteria were applied.

Furthermore, HER2 scoring in gynecologic carcinomas is indication-specific, and current College of American Pathologists (CAP) recommendations provide separate algorithms according to the intended HER2-directed therapy [22,42,45]. For trastuzumab eligibility in endometrial carcinoma, CAP refers to the criteria derived from the randomized phase II trial NCT01367002 in uterine serous carcinoma [45,46]. In this algorithm, intense complete or basolateral/lateral membranous staining in >30% of tumor cells is classified as HER2 3+, whereas HER2 2+ includes intense staining in ≤30% or weak-to-moderate staining in ≥10% of tumor cells. Tumors with an equivocal IHC 2+ score undergo reflex ISH to establish ERBB2 amplification for trastuzumab eligibility [45].

By contrast, DESTINY-PanTumor02 evaluated T-DXd in tumors with HER2 IHC 2+ or 3+ expression using gastric-type scoring criteria, without requiring ERBB2 amplification for IHC 2+ tumors [22,31,42]. Exploratory biomarker analyses from DESTINY-PanTumor02 showed that responses to T-DXd occurred in tumors both with and without tissue HER2 amplification, indicating that HER2 amplification by ISH/FISH is not a prerequisite for clinical activity; however, the small subgroup sizes preclude firm conclusions as to whether amplification enriches for greater treatment benefit [47].

Thus, the different cut-offs reflect treatment- and trial-specific eligibility criteria rather than universally interchangeable definitions of HER2 positivity [31,45].

In our study, however, this issue is unlikely to have had a major impact on the principal findings, as most cases fell into the HER2-zero, HER2-ultralow, or HER2 1+ categories, whereas truly HER2-IHC 2+/3+ tumors were uncommon. Accordingly, for the purposes of our analysis, the more relevant distinction was that between HER2-zero, HER2-ultralow, and HER2 1+, for which breast and gastric scoring systems are largely comparable in surgical specimens.

Our study was not designed to determine eligibility for either trastuzumab or T-DXd but to characterize HER2 expression across the complete low-expression spectrum in EC and HGSOC. We therefore selected a breast cancer-based scoring framework, which permits the distinction between complete absence of membranous staining, IHC 0 with membrane staining (HER2-ultralow), and HER2 1+ [19,22]. Because the metastatic cohort included both resection and biopsy specimens, application of the gastric-type algorithm would additionally have introduced specimen-specific scoring criteria, particularly the tumor-cell-cluster rule for biopsy material. Notably, gastric-type scoring applies different criteria to surgical and biopsy specimens: while surgical specimens are classified according to the proportion of tumor cells showing the respective staining pattern, biopsy specimens may be classified based on a cluster of at least five stained tumor cells, irrespective of the overall percentage of positive tumor cells [22,44]. To maintain internal comparability, the same breast cancer-based scoring algorithm was applied to all primary and metastatic samples. Accordingly, the HER2 scores reported in this study should be interpreted as research-based expression categories and not as direct classifications of eligibility for trastuzumab or T-DXd.

In breast cancer-based scoring, HER2 1+ is defined as faint or barely perceptible incomplete membranous staining in >10% of tumor cells, whereas HER2-zero includes cases with no staining or faint, incomplete membranous staining in ≤10% of tumor cells. HER2-ultralow represents a further subclassification within this HER2-zero category, describing tumors with very faint incomplete membrane staining that does not exceed the threshold for HER2 1+. In gastric cancer scoring of surgical specimens, HER2 1+ is likewise defined by faint or barely perceptible membranous reactivity in part of the membrane, but using a ≥10% threshold, whereas HER2-zero is defined as no reactivity or membranous reactivity in <10% of tumor cells. Thus, at the lower end of the HER2 expression spectrum, the practical distinction between HER2-zero and HER2 1+ is broadly comparable between breast and gastric scoring and mainly depends on weak incomplete membranous staining around the 10% threshold [43,48].

This is important for interpreting this studies results. While the choice of breast versus gastric scoring may have greater impact on the classification of HER2 2+ and HER2 3+ tumors, our central observation concerns the HER2-zero/ultralow/1+ range. For this low-expression spectrum, the distinction is less dependent on fundamentally different staining patterns and more dependent on whether very faint incomplete membranous staining is absent, present below the 10% threshold, or present above this threshold. Therefore, the identification of a substantial HER2-ultralow subgroup in EC and HGSOC is unlikely to be fully explained solely by differences between breast and gastric scoring systems. Rather, it highlights the need to specifically report very low-level HER2 expression instead of grouping all IHC-0 tumors together as HER2-negative.

Nevertheless, concordance between breast and gastric HER2 IHC scoring in endometrioid endometrial adenocarcinomas seems to be high for clearly positive (96.1%) and negative (85.2%) cases in the study of Furuya et al. In contrast, agreement is substantially lower for equivocal tumors (score 2+ assessed analogous to breast cancer), with a concordance rate of only 50.8%. Most discordant cases are classified as negative by breast criteria but equivocal by gastric criteria, highlighting differences in borderline interpretations [49]. However, if one assumes that HER2-targeted therapy may also be effective in tumors classified as HER2-low or ultralow, these discrepancies in scoring become less clinically relevant. Furthermore, HER2-ultralow tumors were not specifically evaluated in this context, which limits direct comparison with our study.

This is especially relevant when interpreting DESTINY-PanTumor02 [31]. As discussed in a recent gynecologic pathology commentary, the trial used a HER2 framework that was not derived from the breast HER2-low/HER2-ultralow paradigm; rather, biomarker eligibility in that study rested on HER2 IHC 2+ or 3+ and has been described as using gastric-type criteria with a 10% threshold, without reflex fluorescence in situ hybridization (FISH) as part of trial eligibility [50]. Moreover, the recent FDA tumor-agnostic approval based on DESTINY-PanTumor02 and companion datasets applies only to IHC 3+ solid tumors. Thus, DESTINY-PanTumor02 does not provide direct evidence for treating HER2-ultralow endometrial or ovarian carcinomas [31].

Our findings therefore support two parallel conclusions. First, the high prevalence of HER2-ultralow expression in our cohorts suggests that this category should not be ignored in gynecologic oncology, especially in the era of ADCs. Second, the current evidence is still insufficient to justify a direct therapeutic transfer of breast-cancer HER2-ultralow concepts to ovarian or endometrial cancer. At present, HER2-ultralow expression in gynecologic tumors should be regarded as a promising exploratory biomarker, not yet as an established treatment-defining category. Prospective trials specifically designed for gynecologic cancers are needed to determine whether HER2-ultralow expression predicts benefit from T-DXd or related agents.

Our survival analyses suggest a potential association between HER2 expression patterns and clinical outcome, particularly regarding PFS. In both endometrial and ovarian carcinoma, patients with HER2-zero tumors demonstrated the most favorable PFS in the Kaplan–Meier analyses, whereas higher HER2 expression categories tended to be associated with shorter PFS. In endometrial carcinoma, HER2 3+ expression was significantly associated with impaired PFS in the univariable Cox regression analysis but did not show any association with PFS in the multivariate analysis. Similarly, in ovarian carcinoma, HER2 1+ expression showed a strong association with reduced PFS. 

The survival findings were additionally examined using Firth’s penalized-likelihood Cox regression because of the small sample size in some HER2 subgroups. The penalized models generally produced estimates similar to those obtained using conventional Cox regression, suggesting that the overall pattern of the results was not solely caused by numerical instability of the conventional models. Nevertheless, Firth penalization cannot compensate for the limited clinical information provided by categories containing only one or two patients.

These observations are in line with previous reports showing that HER2-positive endometrial carcinomas are frequently associated with aggressive clinicopathological characteristics, including the p53-abnormal molecular subtype, serous histology, and high-risk disease categories, while HER2 expression itself was not independently associated with survival after adjustment for established prognostic factors [51]. Additionally, in ovarian carcinoma, HER2-positive tumors were shown to be associated with poorer overall survival, particularly in HRD-associated high-grade serous carcinomas [52].

Interestingly, HER2-ultralow tumors did not demonstrate a significantly worse prognosis compared to HER2 zero tumors in our cohorts. This observation may suggest that HER2-ultralow expression is not primarily a prognostic biomarker. Whether it may serve as a predictive biomarker for HER2-directed ADC therapy remains unknown and requires prospective evaluation. A similar conceptual distinction has emerged in breast cancer, where HER2-low and HER2-ultralow classifications currently appear to be primarily established as treatment-stratifying biomarkers, while their independent prognostic relevance remains incompletely defined [53,54]. Nevertheless, given the retrospective design and limited sample size of our study, definitive conclusions regarding the prognostic role of HER2-ultralow expression in endometrial and ovarian carcinoma cannot yet be drawn. Larger prospective studies with standardized HER2 assessment are required to clarify whether HER2-ultralow disease constitutes a biologically distinct subgroup with specific prognostic or therapeutic implications.

The bivariate analyses did not indicate a consistent clinicopathological phenotype associated with HER2 expression in either EC or HGSOC. Although individual associations were observed, including associations with pM status in primary EC, FIGO stage in metastatic EC lesions, and residual tumor status in metastatic HGSOC lesions, most clinicopathological variables were not significantly associated with HER2 expression.

In EC, the absence of a consistent HER2-associated clinicopathological phenotype should also be interpreted in light of the histological composition of our cohort, which included only very few serous carcinomas, although HER2 alterations have been most frequently reported in serous or p53-mutated endometrial cancer [55]. Given the small number of HER2-positive cases and the limited number of matched metastatic samples, these findings should be interpreted as exploratory. Overall, they suggest that HER2 expression in EC and HGSOC is heterogeneous and may be more relevant as a therapeutic biomarker than as a marker of a distinct clinicopathological subgroup.

The paired comparison of primary tumors and matched metastatic lesions adds an important layer to the interpretation of HER2 expression in EC and HGSOC. In EC, HER2 expression differed between primary and metastatic tissue in a substantial proportion of cases: 11 of 23 paired cases showed lower HER2 expression in the metastatic lesion, seven cases remained unchanged, and five cases showed higher expression in metastatic tissue. In HGSOC, HER2 expression appeared more stable, with 10 of 18 paired cases remaining unchanged, while 4 cases showed lower and 4 cases showed higher HER2 expression in metastatic lesions. Although these findings demonstrate relevant case-level discordance, paired statistical analyses did not indicate a significant systematic shift in HER2 expression between primary tumors and metastases in either tumor entity. Thus, our data suggest that HER2 expression may change during metastatic progression in individual patients, but they do not support a uniform direction of change across the whole cohort.

The clinical relevance of this observation is supported by the breast cancer literature, where receptor discordance between primary tumors and metastatic lesions is well established. Yeung et al. reviewed discordance of HER2 status between primary breast cancer and their metastasis with a median discordance rate of 10% (range 0–44%, IQR 4–17%) in 41 reviewed studies. Loss of HER2 expression was observed in 7.27% of cases, whereas HER2 gain occurred in 6.91%. Moreover, the highest discordance rate was observed in bone metastases [56].

If HER2 discordance is influenced by the anatomical site of metastasis, this aspect cannot be adequately assessed in the present cohort. Bone metastases, which have been discussed as a site with particularly high receptor discordance in other tumor entities, are uncommon in EC and HGSOC and were not sufficiently represented in our dataset. Therefore, no meaningful site-specific analysis of HER2 changes could be performed [57,58]. The limited number of matched metastatic lesions in this study did not allow meaningful site-specific subgroup analyses of HER2 changes.

In a systematic review and meta-analysis including 39 studies, Schrijver et al. confirmed that conversion of ER, PR, and HER2 status occurs during the course of metastatic breast cancer and concluded that reassessment of receptor status in metastatic disease should be strongly encouraged [59].

Although these data originate from breast cancer and therefore cannot be directly extrapolated to gynecologic malignancies, they provide an important conceptual framework of interpreting HER/neu discordance as a clinically relevant phenomenon rather than a purely technical observation.

For endometrial carcinoma, our findings are consistent with previous evidence showing spatial heterogeneity and primary-metastatic discordance of HER2 expression. In a study of paired primary and metastatic endometrial carcinomas, Halle et al. reported discordant HER2 expression patterns and described a substantial reduction of HER2 expression from primary to metastatic disease [60]. This corresponds to our findings.

Although HER2-ultralow expression was not specifically considered, HER2 status was assessed using breast cancer-based scoring criteria, analogous to our study. This methodological overlap provides a relevant basis for comparison with our EC cohort.

A specific feature of our study is the separate assessment of HER2-ultralow expression. This category has not been systematically addressed in previous paired primary–metastatic studies of EC. In our EC cohort, HER2-ultralow primary tumors showed a heterogeneous pattern: only three of 12 HER2-ultralow primary tumors remained HER2-ultralow in the metastatic lesion, whereas seven of 12 shifted to HER2-zero and two of 12 shifted to HER2 1+. Thus, most HER2-ultralow EC primary tumors did not retain the same low-level expression category in matched metastatic tissue. This observation extends previous reports of HER2 loss in metastatic EC by showing that discordance also occurs within the lower HER2 expression spectrum.

In HGSOC, the direct literature on paired HER2 score transitions between primary tumors and metastatic lesions is limited. Nikas et al. analyzed paired primary HGSOC lesions and peritoneal cavity metastases for several biomarkers, including HER2, and reported no HER2 discordance between paired samples [61]. However, HER2-ultralow expression was not evaluated as a separate category in that study.

In our HGSOC cohort, all four HER2-ultralow primary tumors shifted to HER2-zero in the metastatic lesion, whereas four HER2-zero primary tumors shifted to HER2-ultralow. No transitions involving HER2 1+ or HER2 3+ were observed, and one HER2 2+ case remained stable. These findings suggest that HER2 discordance in HGSOC may predominantly occur at the lower end of the expression spectrum.

To our knowledge, the behavior of HER2-ultralow primary tumors during metastatic progression has not yet been specifically reported for HGSOC. Existing HGSOC studies have either focused on HER2 positivity in peritoneal dissemination or compared HER2 expression using different antibodies, rather than evaluating paired HER2-zero, HER2-ultralow, and HER2-low transitions. For example, Yeo et al. demonstrated that HER2 expression can be detected in a subset of peritoneally disseminated HGSOC and that HER2 positivity rates vary depending on the antibody used. These findings underline the technical and biological complexity of HER2 assessment in HGSOC, but they still do not address HER2-ultralow switching between matched primary and metastatic sites [62].

The underlying mechanisms driving HER2 discordance remain incompletely understood. Potential explanations include differences in assay performance, intratumoral and intertumoral heterogeneity, as well as true biological changes during tumor progression or under therapeutic pressure, as suggested by Yeung et al. [56].

Despite the exploratory nature of these findings, they provide preliminary support for an approach that is already clinically relevant in biomarker-driven oncology: HER2 expression should be reassessed in metastatic or recurrent disease when HER2-directed treatment is being considered. This is particularly important because HER2 status may differ between primary and metastatic lesions, and treatment decisions based solely on archival primary tumor tissue may not fully reflect the current disease biology. This appears particularly important in the HER2-low and HER2-ultralow spectrum, where subtle changes in expression may affect treatment eligibility as novel HER2-targeted strategies continue to expand beyond conventionally HER2-positive tumors. However, prospective studies with standardized HER2-ultralow scoring, full-section validation, and paired primary-metastatic sampling are needed to determine whether reassessment of HER2 expression in metastatic gynecologic tumors improves the identification of patients who may benefit from emerging HER2-directed treatment strategies.

This study has several limitations. It is a retrospective single-center analysis with limited sample size, especially for metastatic lesions and for the rare HER2-positive categories. The use of tissue microarrays may underestimate intratumoral heterogeneity compared with full-section analysis, which is particularly relevant for HER2. However, to better account for intratumoral heterogeneity, each tumor was sampled with four cores, including two from the tumor center and two from peripheral tumor areas.

Despite these limitations, our study provides clinically relevant data. It shows that in endometrial carcinoma and HGSOC, the HER2 landscape is dominated not by classical HER2 positivity but by HER2-zero and HER2-ultralow expression. This observation may help refine future trial design. Rather than restricting enrollment only to HER2 IHC 3+ cases, future gynecologic studies should consider stratified inclusion of HER2-ultralow and HER2-low populations, ideally with tumor-type-specific pathology review and harmonized scoring algorithms. Such an approach may clarify whether the therapeutic success of HER2-directed ADCs can be expanded to a broader population of women with gynecologic cancer. Prospective, gynecologic tumor-specific studies are therefore required before HER2-ultralow can be used as a treatment-guiding biomarker in routine practice.

## 5. Conclusions

In conclusion, this study demonstrates that HER2 expression in EC and HGSOC is characterized predominantly by HER2-zero and HER2-ultralow tumors, whereas classical HER2 3+ expression is rare. By specifically separating HER2-zero from HER2-ultralow disease, our data identify a relevant subgroup of patients that would remain largely unrecognized by a purely binary HER2-positive versus HER2-negative classification. This distinction may become increasingly important as HER2-directed antibody–drug conjugates continue to expand the therapeutic relevance of low-level HER2 expression across solid tumors.

In addition, our paired analysis of primary tumors and matched metastatic lesions shows that HER2 expression is not necessarily stable during metastatic progression. Discordance was particularly frequent in EC and also detectable in HGSOC, mainly within the low and ultralow expression spectrum. However, the clinical relevance of HER2-ultralow expression in gynecologic malignancies remains to be established. Based on our findings, we hypothesize that HER2-ultralow may represent a clinically relevant subgroup in EC and HGSOC, particularly as HER2-directed antibody–drug conjugates expand the therapeutic relevance of low-level HER2 expression. Prospective studies with standardized HER2 scoring, validation in full-section specimens, and systematic assessment of matched primary and metastatic tissue are needed to determine whether HER2-ultralow expression has predictive value for HER2-directed therapies in gynecologic cancers.

## Figures and Tables

**Table 1:** Clinicopathological characteristics and HER2 Scores in endometrial and ovarian carcinomas.

Characteristic	Endometrial Carcinoma *n* = 117 (Primary Tumor) *n* = 23 (Metastasis)	Ovarian Carcinoma *n* = 52 (Primary Tumor) *n* = 18 (Metastasis)
**HER2 Score of the primary tumor, *n* (%)**		
Zero	58 (49.6%)	38 (73.1%)
Ultralow	49 (41.9%)	11 (21.2%)
HER2 Score 1+	7 (6.0%)	1 (1.9%)
HER2 Score 2+	1 (0.9%)	1 (1.9%)
HER2 Score 3+	2 (1.7%)	1 (1.9%)
**HER2 Score of the metastasis, *n* (%)**		
Zero	12 (52.2%)	13 (72.2%)
Ultralow	7 (30.4%)	4 (22.2%)
HER2 Score 1+	3 (13.0%)	0 (0%)
HER2 Score 2+	1 (4.3%)	1 (5.6%)
HER2 Score 3+	0 (0%)	0 (0%)
**Histological type, *n* (%)**		
Endometrioid carcinoma	112 (95.7%)	NA
Other histological subtype	5 (4.3%)	NA
**Age, Mean (Median, Min–Max)**	67 (67, 41–91)	64 (65, 44–87)
**FIGO, *n* (%)**		
I	75 (64.1%)	4 (7.7%)
II	9 (7.7%)	7 (13.5%)
III	23 (19.7%)	25 (48.1%)
IV	10 (8.5%)	16 (30.8%)
**pT, *n* (%)**		
pT1	83 (70.9%)	4 (7.7%)
pT2	13 (11.1%)	8 (15.4%)
pT3	20 (17.1%)	40 (77%)
pT4	1 (0.9%)	0 (0%)
**pN, *n* (%)**		
pN0	37 (31.6%)	18 (34.6%)
pN1	21 (17.9%)	23 (44.2%)
pNx	59 (50.4%)	11 (21.2%)
**pM, *n* (%)**		
pM0	93 (79.5%)	36 (69.2%)
pM1	24 (20.5%)	16 (30.8%)
**L, *n* (%)**		
L0	87 (74.4%)	28 (53.8%)
L1	30 (25.6%)	24 (46.2%)
**V, *n* (%)**		
V0	108 (92.3%)	48 (92.3%)
V1	9 (7.7%)	4 (7.7%)
**Pn, *n* (%)**		
Pn0	117 (100%)	51 (98.1%)
Pn1	0 (0%)	1 (1.9%)
**R, *n* (%)**		
R0	103 (88%)	43 (82.7%)
R1	9 (7.7%)	7 (13.5%)
Rx	5 (4.3%)	2 (3.8%)
**Progression-Free Survival in Months, Mean (Median, Min–Max)**	68 (57, 1–206)	28 (16, 1–129)
**Progression, *n* (%)**	59 (50%)	49 (94%)

Data are presented as *n* (%) or mean (median, range), as appropriate. Percentages may not sum to 100% due to rounding. HER2 scores were categorized as zero, ultralow, 1+, 2+, and 3+. Matched metastatic tissue was available for 23 EC and 18 HGSOC cases. NA: not applicable.

**Table 2:** Univariable and multivariable Cox regression analysis of progression-free survival in endometrial carcinoma.

	Absolute	Univariable				Multivariable			
Characteristic	*N* = 117 ^1^	*N*	HR ^2^	95% CI ^2^	*p*-Value	*N*	HR ^2^	95% CI ^2^	*p*-Value
**HER2 Score**		117				117			
Zero	58 (50%)		—	—			—	—	
Ultralow	49 (42%)		1.39	0.81, 2.39	0.2		1.01	0.56, 1.81	>0.9
HER2 Score 1+	7 (6.0%)		1.21	0.36, 4.02	0.8		0.75	0.17, 3.29	0.7
HER2 Score 2+	1 (0.9%)		5.89	0.78, 44.8	0.087		1.90	0.20, 17.7	0.6
HER2 Score 3+	2 (1.7%)		6.44	1.49, 27.8	0.013		1.91	0.30, 12.3	0.5
**Age**	67 (59, 74)	117	1.05	1.03, 1.08	<0.001	117	1.08	1.05, 1.12	<0.001
**Histology**		117							
Endometrioid	112 (96%)		—	—					
Other	5 (4.3%)		1.14	0.36, 3.64	0.8				
**FIGO**		117							
I	75 (64%)		—	—					
II	9 (7.7%)		2.07	0.80, 5.38	0.13				
III	23 (20%)		2.65	1.41, 4.97	0.002				
IV	10 (8.5%)		6.59	3.15, 13.8	<0.001				
**pT**		117				117			
pT1	83 (71%)		—	—			—	—	
pT2	13 (11%)		2.46	1.13, 5.33	0.023		3.68	1.38, 9.82	0.009
pT3	20 (17%)		2.88	1.57, 5.30	<0.001		2.53	1.00, 6.43	0.051
pT4	1 (0.9%)		3.58	0.48, 26.5	0.2		1.02	0.07, 15.0	>0.9
**pN**		117				117			
pN0	37 (32%)		—	—			—	—	
pN1	21 (18%)		2.01	0.98, 4.14	0.056		0.40	0.14, 1.13	0.084
pNx	59 (50%)		1.16	0.63, 2.13	0.6		1.25	0.63, 2.49	0.5
**pM**		117				117			
0	93 (79%)		—	—			—	—	
1	24 (21%)		3.69	2.15, 6.33	<0.001		4.33	1.78, 10.5	0.001
**L**		117				117			
0	87 (74%)		—	—			—	—	
1	30 (26%)		3.40	1.99, 5.80	<0.001		1.84	0.88, 3.81	0.10
**V**		117							
0	108 (92%)		—	—					
1	9 (7.7%)		1.15	0.49, 2.69	0.8				
**Pn**									
0	117 (100%)								
R		117				117			
0	103 (88%)		—	—			—	—	
1	9 (7.7%)		2.47	1.11, 5.49	0.026		1.36	0.40, 4.68	0.6
Rx	5 (4.3%)		3.45	1.36, 8.74	0.009		0.91	0.32, 2.60	0.9

^1^ Data are presented as *N* (%) or median (Q1, Q3). ^2^ HRs are shown with 95% confidence intervals. HER2 score zero served as the reference category. Cox proportional hazards regression models were used to evaluate associations with progression-free survival. Dashes indicate reference categories or could not be calculated.

**Table 3:** Univariable and multivariable Cox regression analysis of progression-free survival in HGSOC.

	Absolute	Univariable				Multivariable			
Characteristic	*N* = 52 ^1^	*N*	HR ^2^	95% CI ^2^	*p*-Value	*N*	HR ^2^	95% CI ^2^	*p*-Value
**HER2 Score**		52				52			
Zero	38 (73%)		—	—			—	—	
Ultralow	11 (21%)		0.71	0.34, 1.47	0.4		0.94	0.44, 2.03	0.9
HER2 Score 1+	1 (1.9%)		47.5	2.96, 762	0.006		34.8	1.95, 619	0.016
HER2 Score 2+	1 (1.9%)		1.22	0.16, 9.08	0.8		1.50	0.20, 11.5	0.7
HER2 Score 3+	1 (1.9%)		0.98	0.13, 7.28	>0.9		1.15	0.15, 8.75	0.9
**Age**	65 (55, 72)	52	1.01	0.98, 1.04	0.5				
**FIGO**		52							
1	4 (7.7%)		—	—					
2	7 (13%)		2.84	0.56, 14.6	0.2				
3	25 (48%)		3.68	0.86, 15.8	0.080				
4	16 (31%)		6.44	1.42, 29.1	0.016				
**pT**		52				52			
pT1	4 (7.7%)		—	—			—	—	
pT2	8 (15%)		3.09	0.62, 15.3	0.2		4.32	0.78, 24.0	0.095
pT3	40 (77%)		4.23	1.00, 17.8	0.049		4.94	1.07, 22.7	0.040
**pN**		52							
pN0	18 (35%)		—	—					
pN1	23 (44%)		1.90	0.97, 3.73	0.060				
pNx	11 (21%)		1.63	0.73, 3.60	0.2				
**M**		52							
pM0	36 (69%)		—	—					
pM1	16 (31%)		1.64	0.89, 3.02	0.12				
**L**		52							
L0	28 (54%)		—	—					
L1	24 (46%)		1.41	0.79, 2.52	0.2				
**V**		52				52			
V0	48 (92%)		—	—			—	—	
V1	4 (7.7%)		2.96	1.00, 8.73	0.049		3.63	1.17, 11.3	0.025
**Pn**		52							
Pn0	51 (98%)		—	—					
Pn1	1 (1.9%)		3.54	0.46, 27.0	0.2				
**R**		52				52			
R0	43 (83%)		—	—			—	—	
R1	7 (13%)		2.40	1.05, 5.45	0.037		2.08	0.84, 5.14	0.11
Rx	2 (3.8%)		3.51	0.80, 15.3	0.095		9.10	1.77, 46.7	0.008

^1^ Data are presented as *N* (%) or median (Q1, Q3). ^2^ HRs are shown with 95% confidence intervals. HER2 score zero served as the reference category. Cox proportional hazards regression models were used to evaluate associations with progression-free survival. Dashes indicate reference categories or could not be calculated.

**Table 4:** Concordance and directional changes of HER2 expression between matched primary tumors and metastatic lesions.

Parameter	EC	HGSOC
Matched pairs, *n*	23	18
Lower in metastasis, *n* (%)	11 (47.8%)	4 (22.2%)
Unchanged, *n* (%)	7 (30.4%)	10 (55.6%)
Higher in metastasis, *n* (%)	5 (21.7%)	4 (22.2%)
Weighted kappa	0.338	0.333
Kappa *p*-value	0.086	0.157
Wilcoxon *p*-value	0.073	1

Data are presented as *n* (%). Directional changes refer to lower, unchanged, or higher HER2 expression in metastatic tissue compared with matched primary tumor tissue. Agreement was assessed using weighted Cohen’s kappa, and paired directional changes were evaluated using the Wilcoxon signed-rank test. Two-sided *p*-values are reported.

## Data Availability

The data presented in this study are available on request from the corresponding author due to institutional and data protection regulations, individual patient data are not publicly available.

## Supplementary Materials

The following supporting information can be downloaded at: https://www.mdpi.com/article/10.3390/cancers18172843/s1, Figure S1: Representative HER2 immunohistochemistry images from primary endometrial carcinoma and primary high-grade serous ovarian carcinoma; Figure S2: Validation of Immunohistochemical Staining Using anonamized HER2-Negative (0) and HER2-Positive (3+) invasive Ductal Carcinoma Control Tissues; Figure S3: HER2-zero to HER2-ultralow transition in HGSOC; Figure S4: HER2-ultralow to HER2-zero transition in HGSOC; Figure S5: Loss of HER2 3+ expression in endometrial carcinoma; Figure S6: HER2-zero to HER2-ultralow transition in endometrial carcinoma; Table S1: Age distribution according to HER2 expression in primary endometrial carcinoma; Table S2: Age distribution according to HER2 expression in primary high-grade serous ovarian carcinoma; Table S3: Univariable and multivariable Firth penalized likelihood Cox regression analyses of progression-free survival in endometrial carcinoma; Table S4: Univariable and multivariable Firth penalized likelihood Cox regression analyses of progression-free survival in high-grade serous ovarian carcinoma; Table S5. Bivariate analysis of clinicopathological characteristics according to HER2/neu expression in primary tumors and metastasis in endometrial carcinoma; Table S6: Bivariate analysis of clinicopathological characteristics according to HER2/neu expression in primary tumors and metastasis in ovarian carcinoma; Table S7. Changes in HER2/neu scores between primary and metastatic tumor tissue for EC; Table S8: Changes in HER2/neu scores between primary and metastatic tumor tissue for OC.

## Abbreviations

The following abbreviations are used in this manuscript:

ADC	Antibody–drug conjugates
CAP	College of American Pathologists
EC	Endometrial carcinoma
FDA	U.S. Food and Drug Administration
FIGO	International Federation of Gynecology and Obstetrics
FISH	Fluorescence in situ hybridization
H&E	Hematoxylin and Eosin
HER	Human Epidermal Growth Factor Receptor 2
HGSOC	High-grade serous ovarian carcinoma
HR	Hormone receptor
ICP	Institute for Clinical Pathology
IHC	Immunohistochemistry
ISH	In situ hybridization
L	Lymphatic vessel invasion
M	Distant metastasis
MMR	Mismatch repair
OC	Ovarian carcinoma
ORR	Overall response rate
OS	Overall survival
PFS	Progressive-free survival
pN	Lymph node invasion, X indicates that status could not be assessed
Pn	Perineural Invasion
R	Residual tumor status
ROI	Region of interest
T-Dxd	Trastuzumab Deruxtecan
TMA	Tissue microarray
TNM	Tumor, nodus, metastase
V	Venous invasion
WHO	Word Health Organization
