# Supplementary material for: Expression of HER2 Ultralow in Endometrial Carcinoma, High-Grade Serous Ovarian Cancer and Their Metastases
Source: Cancers (Basel). 2026 Sep 2;18(17):2843. doi: 10.3390/cancers18172843 (PMC13565059; doi:10.3390/cancers18172843)
Supplement: Supplementary file 1 [file cancers-18-02843-s001.zip › cancers-4458652-supplementary.pdf]

Figure S1(A).

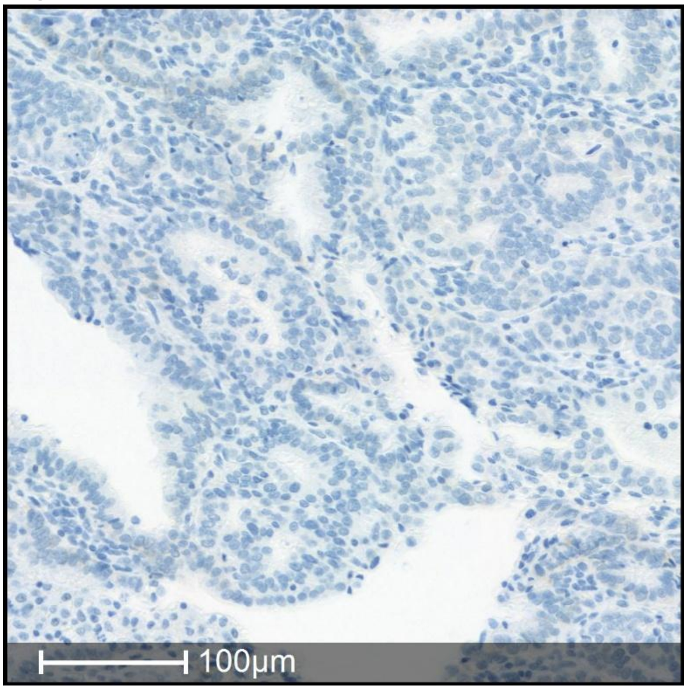

Figure S1(B).

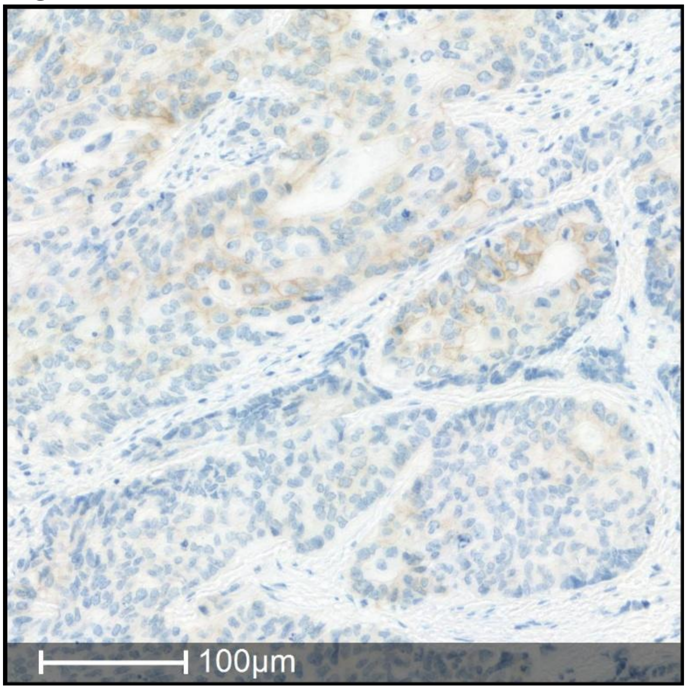

Figure S1(C).

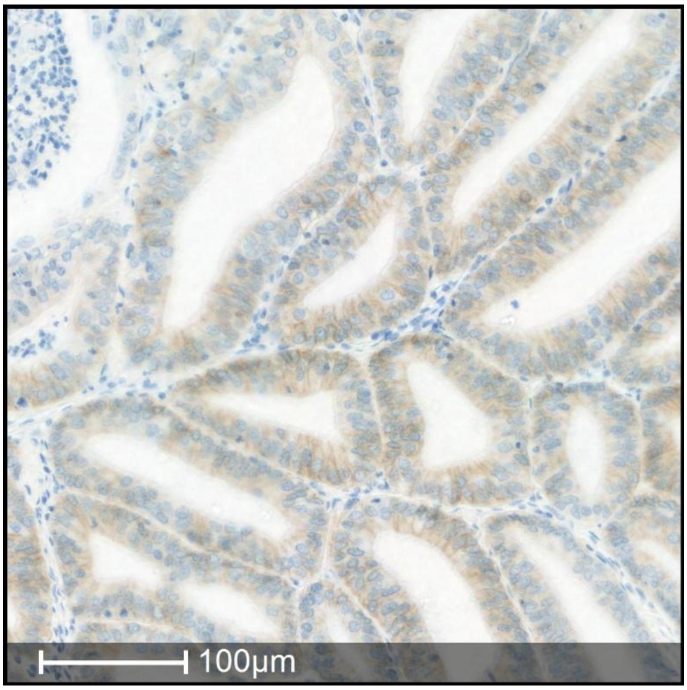

Figure S1(D).

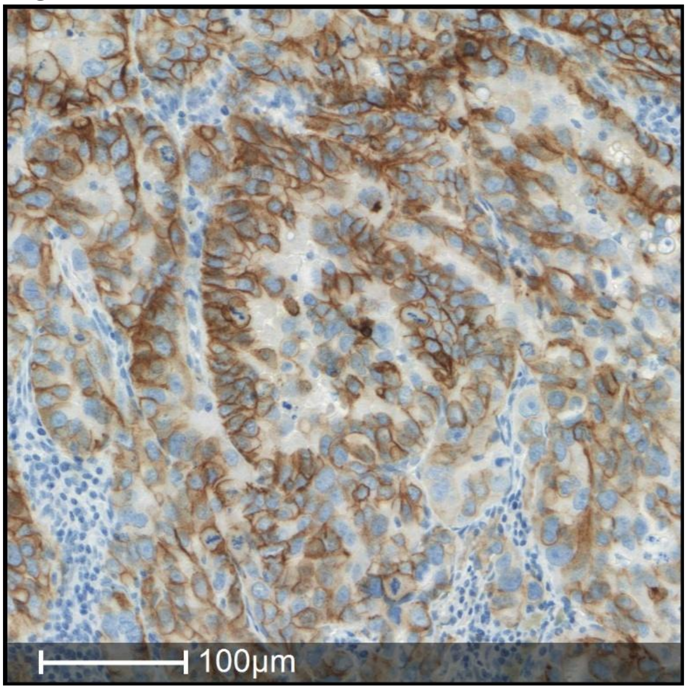

Figure S1(E).

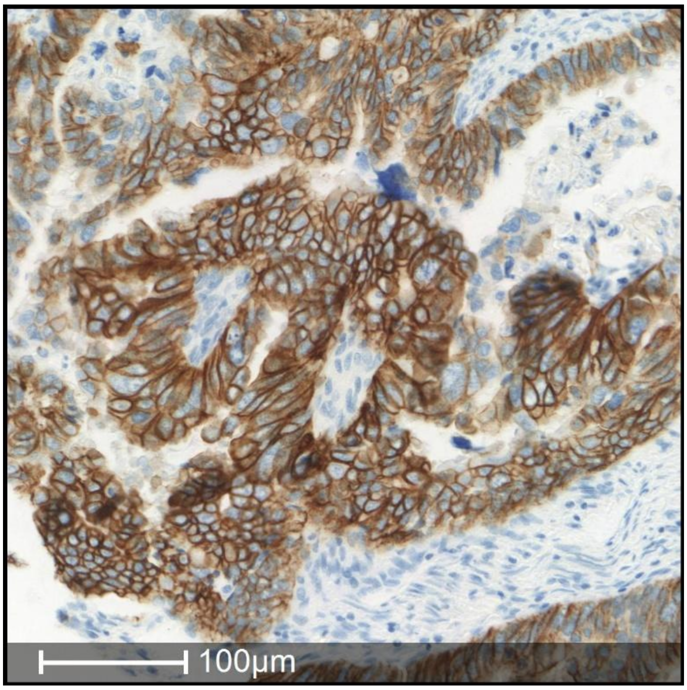

Figure S1(F).

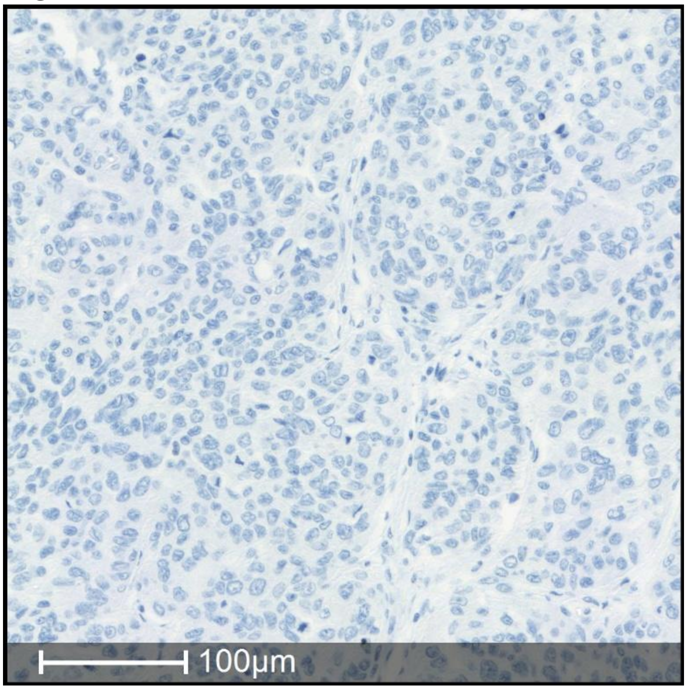

Figure S1(G).

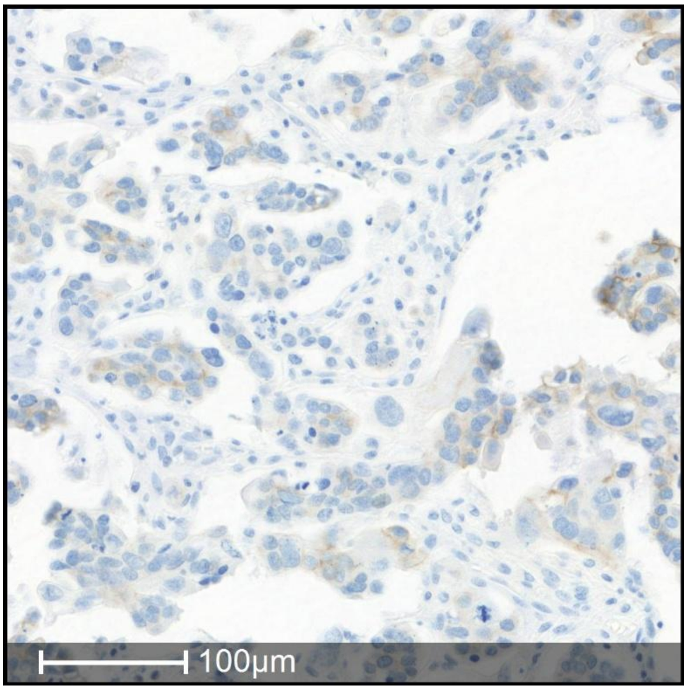

Figure S1(H).

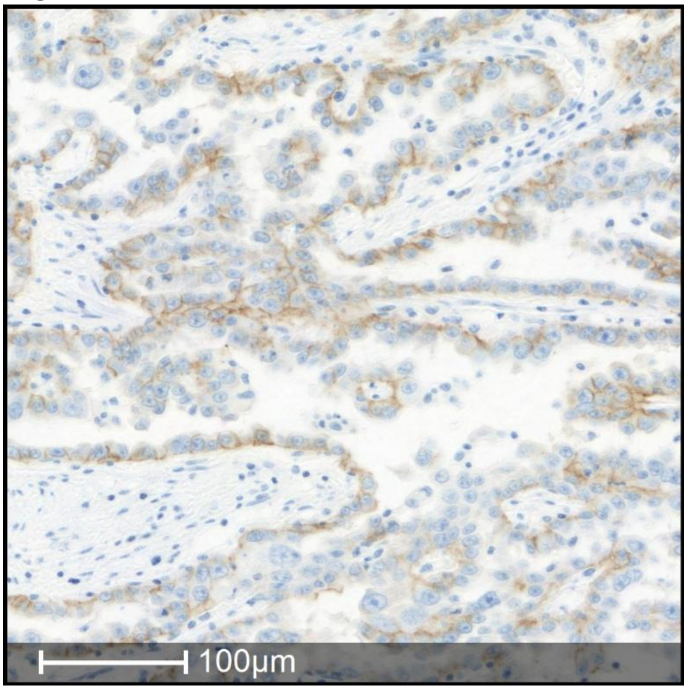

Figure S1(I).

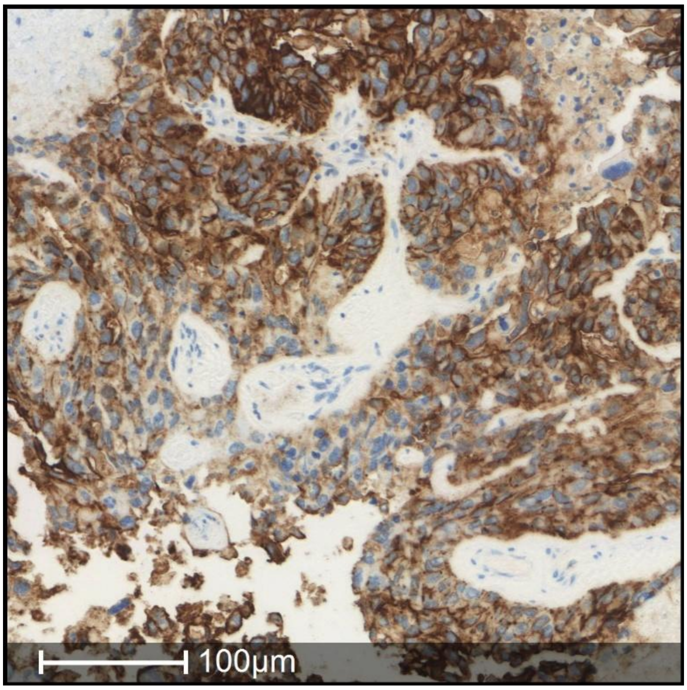

Figure S1(J).

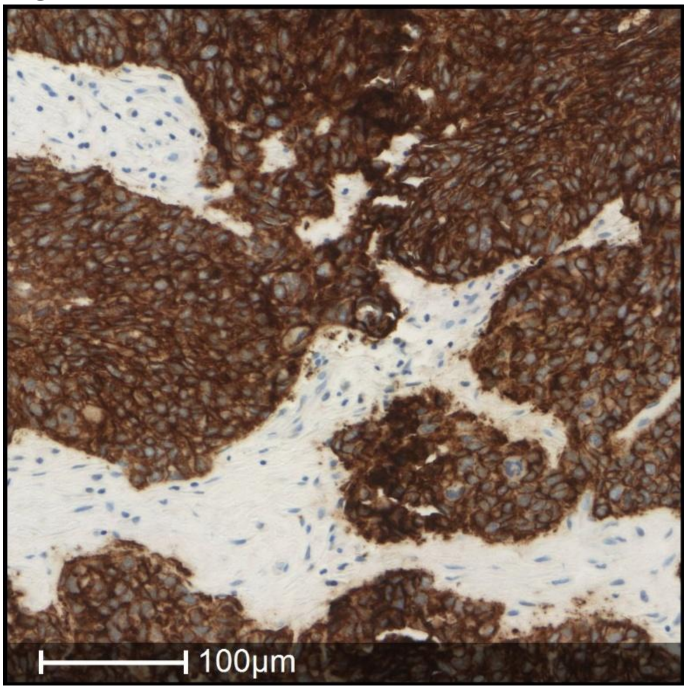

Supplementary Figure S1 (A-J): Representative HER2 immunohistochemistry images from primary endometrial carcinoma and primary high-grade serous ovarian carcinoma. Images were captured from digitally scanned whole-slide images using HALO AI image analysis software. Panels (A–E) show primary endometrial carcinoma with HER2-zero (A), HER2-ultralow (B), HER2 1+ (C), HER2 2+ (D), and HER2 3+ (E) expression. Panels (F–J) show primary high-grade serous ovarian carcinoma with HER2-zero (F), HER2-ultralow (G), HER2 1+ (H), HER2 2+ (I), and HER2 3+ (J) expression.

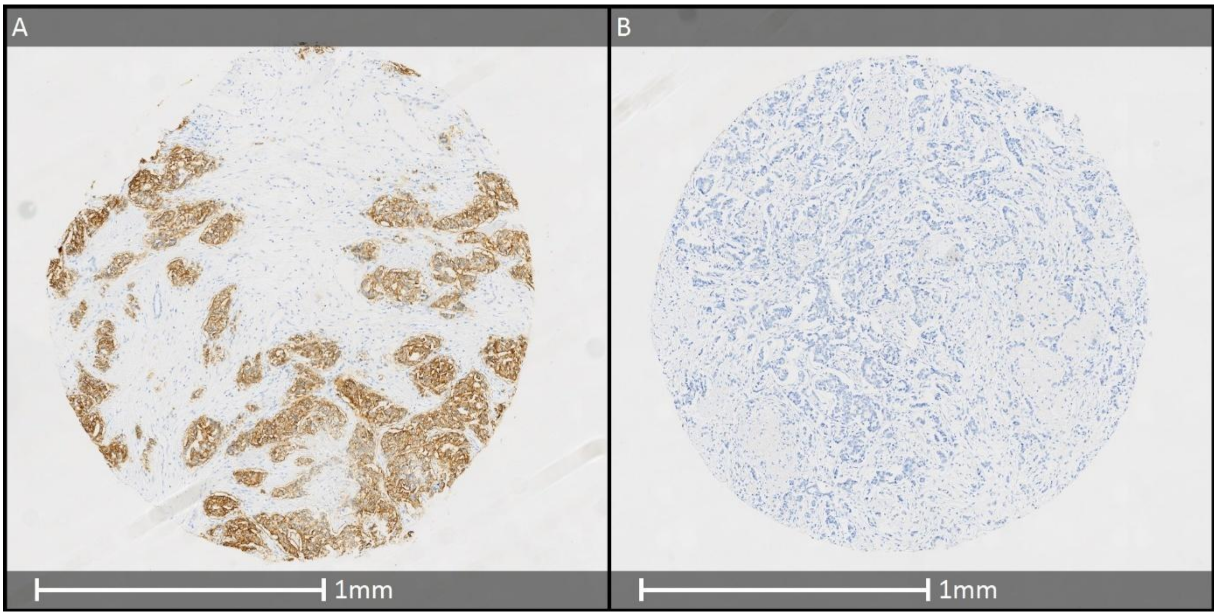

Supplementary Figure S2 (A-B). Validation of Immunohistochemical Staining Using anonamized HER2-Negative (0) and HER2-Positive (3+) invasive Ductal Carcinoma Control Tissues.

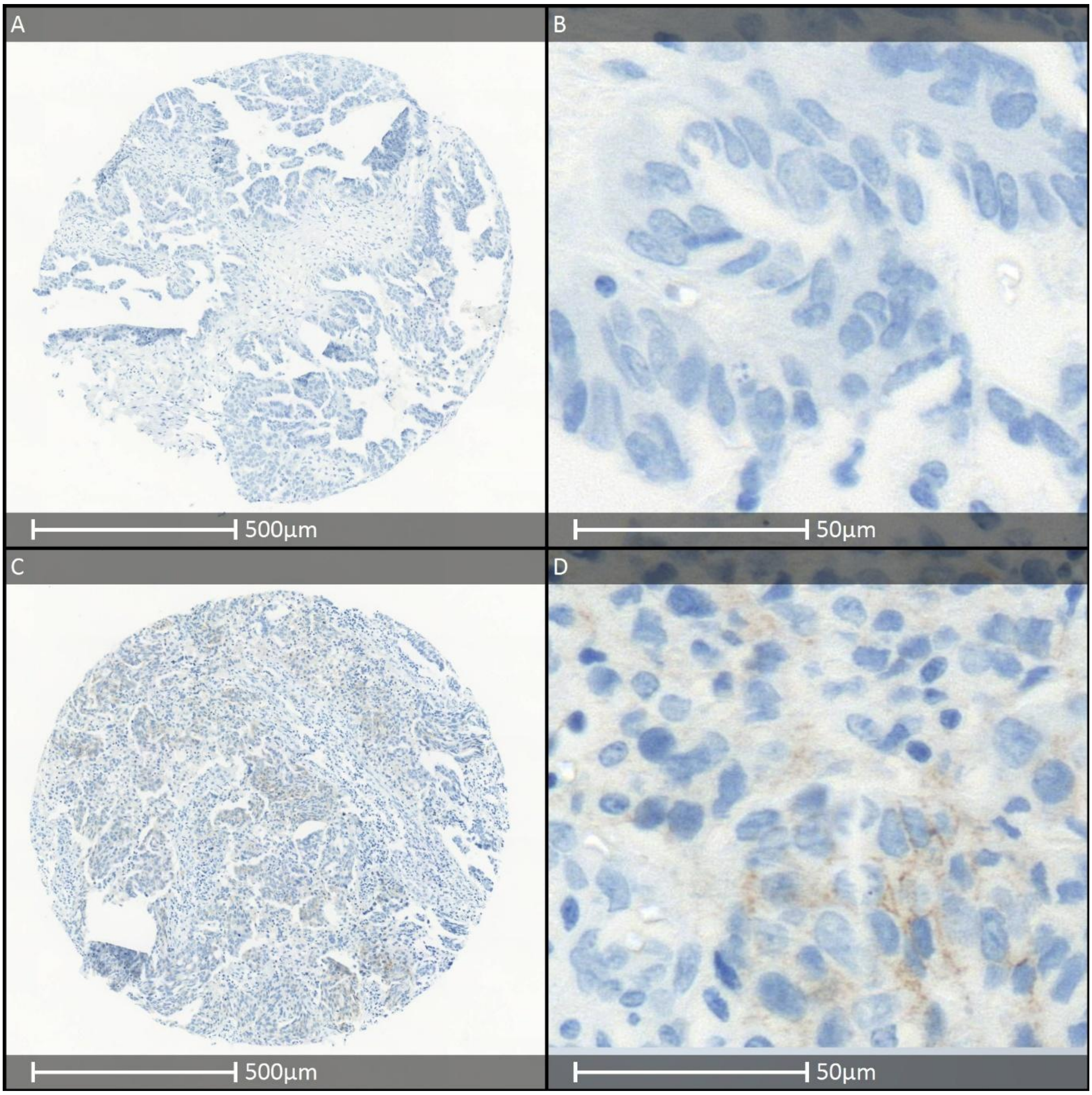

Supplementary Figure S3. HER2-zero to HER2-ultralow transition in HGSOC. HER2 immunohistochemistry of the primary tumor at low (A) and high magnification (B) and the matched extra-regional peribronchial lymph node metastasis at low (C) and high magnification (D). The primary tumor was HER2-zero, whereas the metastasis showed HER2-ultralow expression.

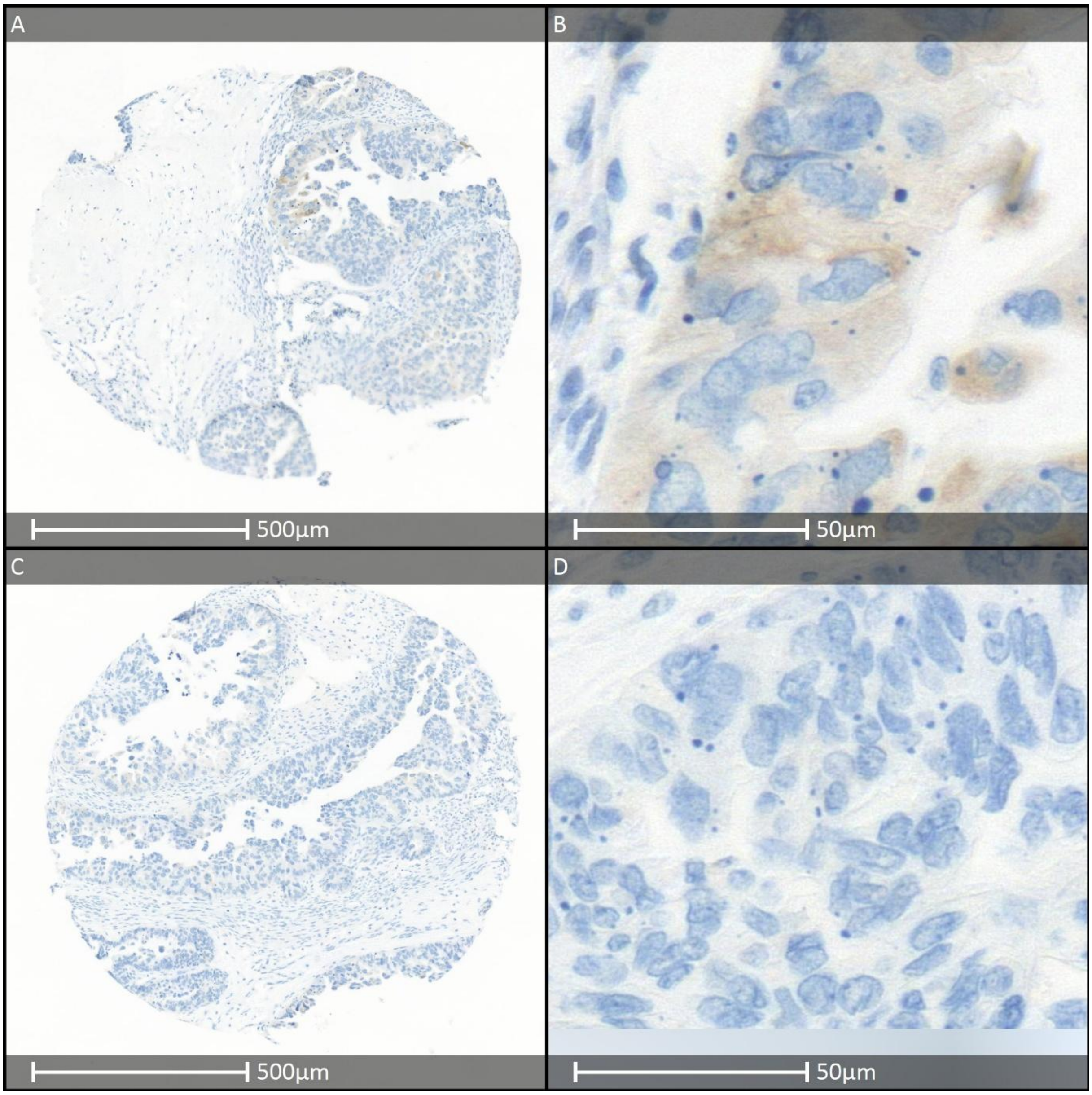

Supplementary Figure S4. HER2-ultralow to HER2-zero transition in HGSOC. HER2 immunohistochemistry of the primary tumor at low (A) and high magnification (B) and the matched omental metastasis at low (C) and high magnification (D). The primary tumor showed HER2-ultralow expression, whereas the metastasis was HER2-zero.

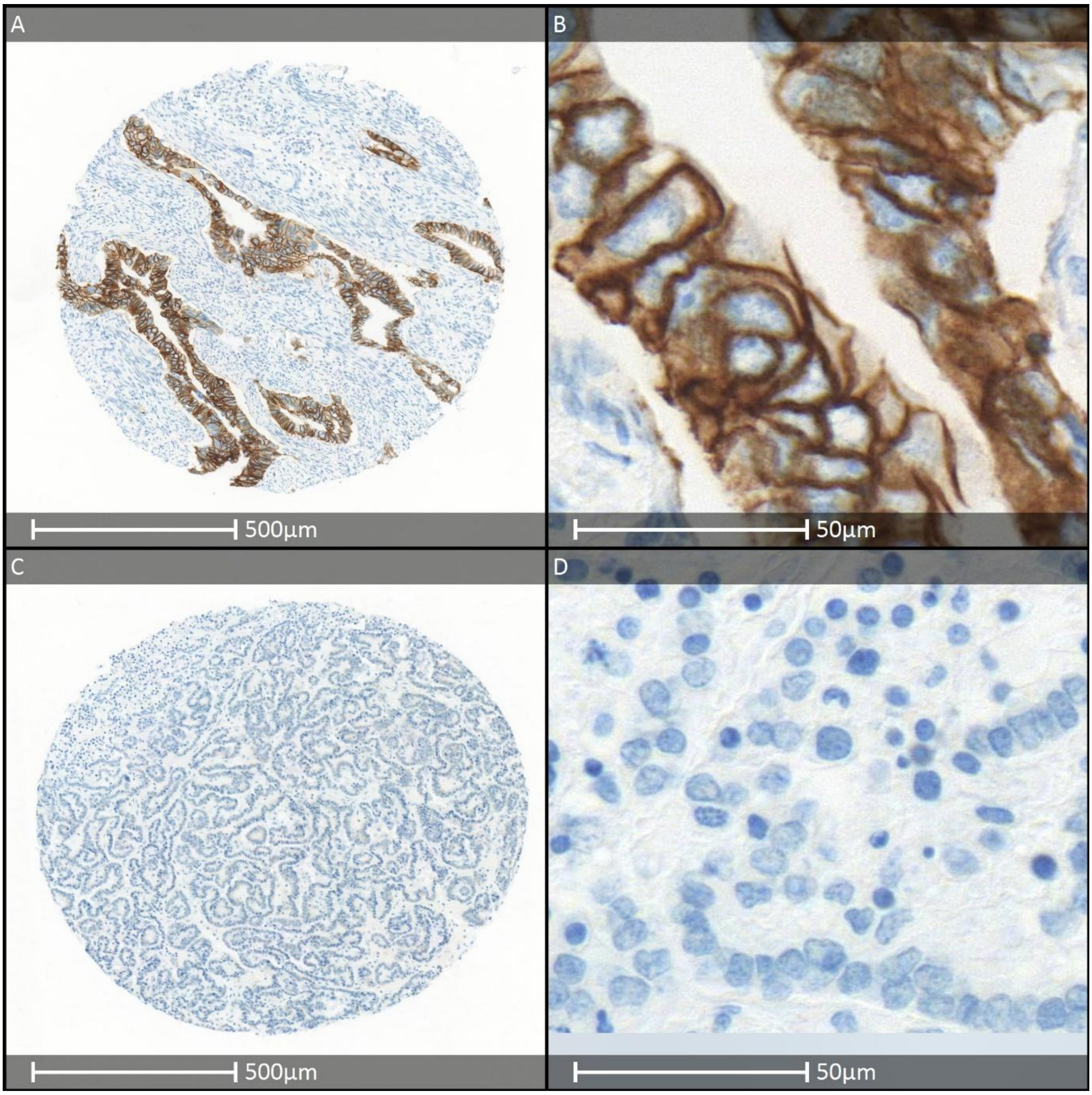

Supplementary Figure S5. Loss of HER2 3+ expression in endometrial carcinoma. HER2 immunohistochemistry of the primary tumor at low (A) and high magnification (B) and the matched sigmoid colon metastasis at low (C) and high magnification (D). The primary tumor showed HER2 3+ expression, whereas the metastasis was HER2-zero.

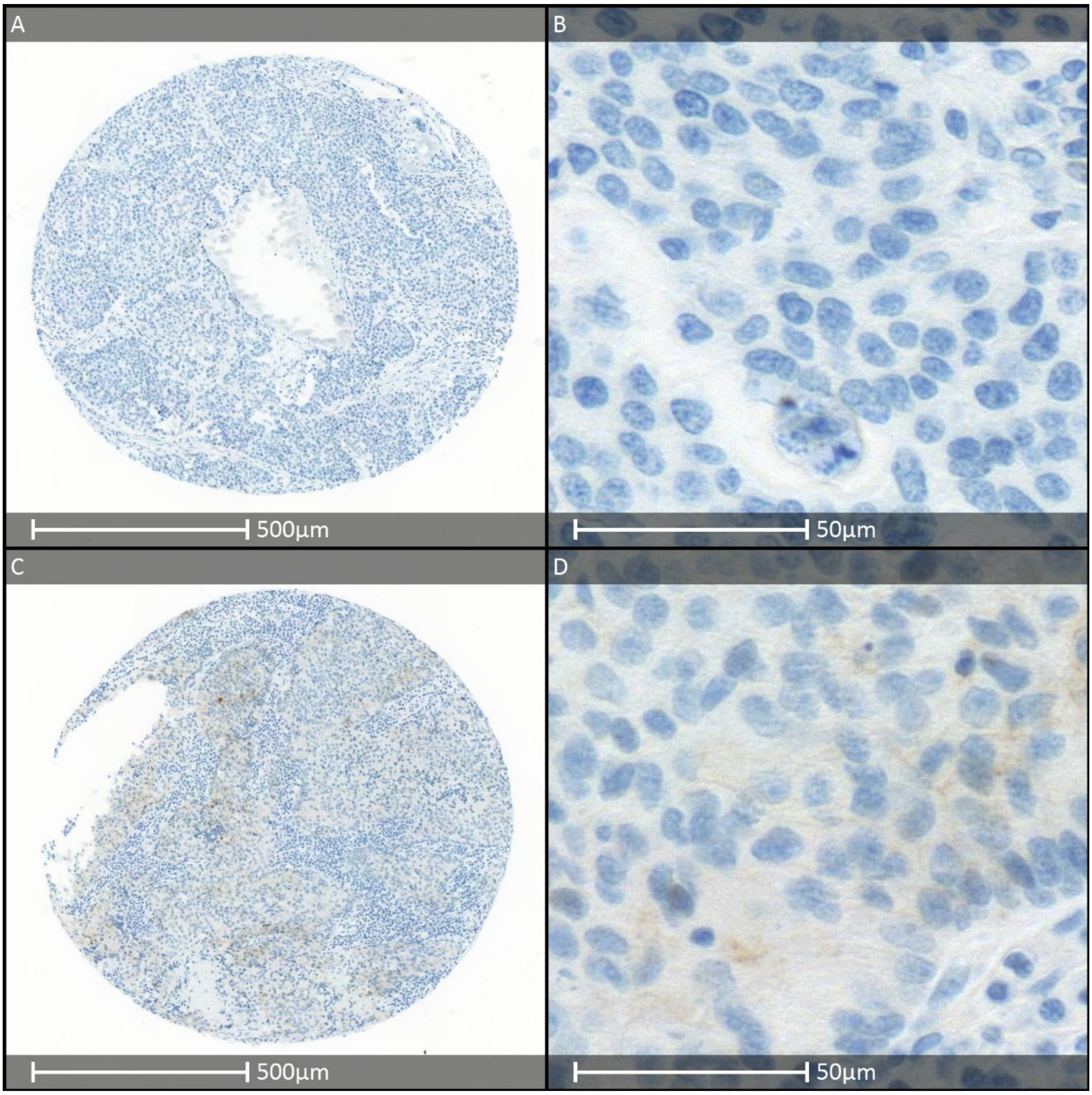

Supplementary Figure S6. HER2-zero to HER2-ultralow transition in endometrial carcinoma. HER2 immunohistochemistry of the primary tumor at low (A) and high magnification (B) and the matched extra-regional paracaval lymph node metastasis at low (C) and high magnification (D). The primary tumor was HER2-zero, whereas the metastasis showed HER2-ultralow expression.

Supplementary Table S1. Age distribution according to HER2 expression in primary endometrial carcinoma

**Age**	66.0 (11.7); 65.0 (43.0-91.0)	67.6 (10.4); 68.0 (41.0-88.0)	70.6 (9.3); 66.0 (63.0-87.0)	64.0 (NA); 64.0 (64.0-64.0)	65.0 (1.4); 65.0 (64.0-66.0)	0.8

Data are presented as: [1] mean (SD) and median (range). Differences in age across HER2 expression categories were assessed using the [2] Kruskal–Wallis rank-sum test. SD, standard deviation; NA, not applicable.

Supplementary Table S2. Age distribution according to HER2 expression in primary high-grade serous ovarian carcinoma

**Variable**	**Zero** N = 38[1]	**Ultralow** N = 11[1]	**HER2 Score 1+** N = 1[1]	**HER2 Score 2+** N = 1[1]	**HER2 Score 3+** N = 1[1]	**p-value**[2]
**Age**	63.2 (11.0); 63.6 (44.0-86.5)	69.7 (8.2); 69.6 (57.6-84.3)	49.0 (NA); 49.0 (49.0-49.0)	70.8 (NA); 70.8 (70.8-70.8)	51.9 (NA); 51.9 (51.9-51.9)	0.2

Data are presented as: 1 mean (SD) and median (range). Differences in age across HER2 expression categories were assessed using the 2 Kruskal–Wallis rank-sum test. SD, standard deviation; NA, not applicable.

Supplementary Table S3. Univariable and multivariable Firth penalized-likelihood Cox regression analyses of progression-free survival in endometrial carcinoma.

Characteristic	Univariable Firth Cox regression N=117		Multivariable Firth Cox regression N=117	
	HR (95% CI)	p-value	HR (95% CI)	p-value
**HER2 score**				
HER2-zero (n=58)	Reference	NA	Reference	NA
HER2-ultralow (n=49)	1.39 (0.81-2.39)	0.227	1.01 (0.57-1.81)	0.972
HER2 1+ (n=7)	1.38 (0.37-3.74)	0.587	0.91 (0.18-2.93)	0.887
HER2 2+ (n=1)	8.59 (0.94-34.62)	0.055	2.43 (0.23-13.65)	0.396
HER2 3+ (n=2)	7.67 (1.53-24.20)	0.018	2.09 (0.31-10.97)	0.420
**Global HER2 effect (4 df)**	NA	0.047	NA	0.856
**Clinicopathological covariates included in the multivariable model**				
Age (per year)	NA	NA	1.08 (1.05-1.12)	<0.001
pT1 (reference)	NA	NA	Reference	NA
pT2	NA	NA	3.76 (1.36-9.41)	0.012
pT3	NA	NA	2.48 (0.94-5.93)	0.066
pT4	NA	NA	1.13 (0.08-11.63)	0.922
pN0 (reference)	NA	NA	Reference	NA
pN1	NA	NA	0.41 (0.14-1.11)	0.079
pNx	NA	NA	1.21 (0.63-2.46)	0.573
pM0 (reference)	NA	NA	Reference	NA

pM1	NA	NA	4.27 (1.73-10.02)	0.002
L0 (reference)	NA	NA	Reference	NA
L1	NA	NA	1.85 (0.88-3.75)	0.101
R0 (reference)	NA	NA	Reference	NA
R1	NA	NA	1.45 (0.42-4.71)	0.546
Rx	NA	NA	0.98 (0.32-2.53)	0.964

HR, hazard ratio; CI, confidence interval; df, degrees of freedom; NA, not applicable or not estimated; PFS, progression-free survival.

Firth penalized-likelihood Cox regression was performed as a sensitivity analysis. HRs are reported with profile penalized-likelihood 95% CIs and profile penalized-likelihood p-values. HER2-zero served as the reference category; no data-driven regrouping of HER2 categories was performed.

The global HER2 p-value was obtained from a 4-df penalized likelihood-ratio test jointly evaluating the HER2-ultralow, HER2 1+, HER2 2+, and HER2 3+ coefficients.

The multivariable model was adjusted for age, pathological tumor stage (pT), pathological nodal status (pN), distant metastasis (pM), lymphatic vessel invasion (L), and residual tumor status (R).

Supplementary Table S4. Univariable and multivariable Firth penalized-likelihood Cox regression analyses of progression-free survival in high-grade serous ovarian carcinoma.

Characteristic	Univariable Firth Cox regression N=52		Multivariable Firth Cox regression N=52	
	HR (95% CI)	p-value	HR (95% CI)	p-value
**HER2 score**				
HER2-zero (n=38)	Reference	NA	Reference	NA
HER2-ultralow (n=11)	0.74 (0.34-1.46)	0.404	0.98 (0.44-1.99)	0.956
HER2 1+ (n=1)	47.86 (3.87-591.34)	0.006	33.06 (2.47-448.37)	0.012
HER2 2+ (n=1)	1.80 (0.20-6.97)	0.520	2.16 (0.24-8.78)	0.420
HER2 3+ (n=1)	1.45 (0.16-5.58)	0.673	1.66 (0.18-6.69)	0.579
**Global HER2 effect (4 df)**	NA	0.059	NA	0.126
**Clinicopathological covariates included in the multivariable model**				
pT1 (reference)	NA	NA	Reference	NA
pT2	NA	NA	3.84 (0.86-23.62)	0.079
pT3	NA	NA	4.17 (1.21-22.29)	0.021
V0 (reference)	NA	NA	Reference	NA
V1	NA	NA	3.56 (1.07-9.71)	0.039
R0 (reference)	NA	NA	Reference	NA
R1	NA	NA	2.12 (0.81-4.79)	0.118
Rx	NA	NA	10.04 (1.75-40.33)	0.014

HR, hazard ratio; CI, confidence interval; df, degrees of freedom; NA, not applicable or not estimated; PFS, progression-free survival.

Firth penalized-likelihood Cox regression was performed as a sensitivity analysis. HRs are reported with profile penalized-likelihood 95% CIs and profile penalized-likelihood p-values. HER2-zero served as the reference category; no data-driven regrouping of HER2 categories was performed.

The global HER2 p-value was obtained from a 4-df penalized likelihood-ratio test jointly evaluating the HER2-ultralow, HER2 1+, HER2 2+, and HER2 3+ coefficients.

The multivariable model was adjusted for age, pathological tumor stage (pT), pathological nodal status (pN), distant metastasis (pM), lymphatic vessel invasion (L), and residual tumor status (R).

Supplementary Table S5. Bivariate analysis of clinicopathological characteristics according to HER2/neu expression in primary tumors and metastasis in endometrial carcinoma.

Variable	HER2/neu Score - Primary							HER2/neu Score Primary - Metastasis						
	N	Zero N = 58[1]	Ultralow N = 49[1]	HER2 Score 1+ N = 7[1]	HER2 Score 2+ N = 1[1]	HER2 Score 3+ N = 2[1]	p-value[2]	N	Zero N = 12[1]	Ultralow N = 7[1]	HER2 Score 1+ N = 3[1]	HER2 Score 2+ N = 1[1]	HER2 Score 3+ N = 0[1]	p-value[2]
Her2_score_metastasis	23						0.2							
Zero		4 (57%)	7 (58%)	0 (0%)	0 (0%)	1 (50%)								
Ultralow		3 (43%)	3 (25%)	1 (100%)	0 (0%)	0 (0%)								
HER2 Score 1+		0 (0%)	2 (17%)	0 (0%)	1 (100%)	0 (0%)								
HER2 Score 2+		0 (0%)	0 (0%)	0 (0%)	0 (0%)	1 (50%)								
HER2 Score 3+		0 (0%)	0 (0%)	0 (0%)	0 (0%)	0 (0%)								
Age	117	65 (56, 74)	68 (61, 77)	66 (64, 80)	64 (64, 64)	65 (64, 66)	0.8	23	63 (58, 69)	70 (51, 74)	69 (64, 80)	64 (64, 64)	NA (NA, NA)	0.5
FIGO	117						0.2	23						0.024
I		38 (66%)	32 (65%)	5 (71%)	0 (0%)	0 (0%)			0 (0%)	3 (43%)	1 (33%)	0 (0%)	0 (NA%)	

II		6 (10%)	3 (6.1%)	0 (0%)	0 (0%)	0 (0%)			0 (0%)	0 (0%)	0 (0%)	0 (0%)	0 (NA%)	
III		12 (21%)	8 (16%)	1 (14%)	1 (100%)	1 (50%)			4 (33%)	3 (43%)	2 (67%)	1 (100%)	0 (NA%)	
IV		2 (3.4%)	6 (12%)	1 (14%)	0 (0%)	1 (50%)			8 (67%)	1 (14%)	0 (0%)	0 (0%)	0 (NA%)	
pT	117						0.11	23						0.5
pT1		42 (72%)	35 (71%)	5 (71%)	0 (0%)	1 (50%)			4 (33%)	3 (43%)	1 (33%)	0 (0%)	0 (NA%)	
pT2		8 (14%)	4 (8.2%)	0 (0%)	0 (0%)	1 (50%)			1 (8.3%)	1 (14%)	0 (0%)	1 (100%)	0 (NA%)	
pT3		8 (14%)	10 (20%)	1 (14%)	1 (100%)	0 (0%)			7 (58%)	2 (29%)	2 (67%)	0 (0%)	0 (NA%)	
pT4		0 (0%)	0 (0%)	1 (14%)	0 (0%)	0 (0%)			0 (0%)	1 (14%)	0 (0%)	0 (0%)	0 (NA%)	
pN	117						0.14	23						0.8
pN0		20 (34%)	12 (24%)	4 (57%)	0 (0%)	1 (50%)			2 (17%)	3 (43%)	0 (0%)	0 (0%)	0 (NA%)	

pN1		8 (14%)	11 (22%)	0 (0%)	1 (100%)	1 (50%)			8 (67%)	3 (43%)	2 (67%)	1 (100%)	0 (NA%)	
pNx		30 (52%)	26 (53%)	3 (43%)	0 (0%)	0 (0%)			2 (17%)	1 (14%)	1 (33%)	0 (0%)	0 (NA%)	
pM	117						0.006	23						>0.9
0		51 (88%)	36 (73%)	6 (86%)	0 (0%)	0 (0%)			0 (0%)	0 (0%)	0 (0%)	0 (0%)	0 (NA%)	
1		7 (12%)	13 (27%)	1 (14%)	1 (100%)	2 (100%)			12 (100%)	7 (100%)	3 (100%)	1 (100%)	0 (NA%)	
L	117						0.4	23						0.4
0		44 (76%)	36 (73%)	6 (86%)	0 (0%)	1 (50%)			5 (42%)	4 (57%)	0 (0%)	0 (0%)	0 (NA%)	
1		14 (24%)	13 (27%)	1 (14%)	1 (100%)	1 (50%)			7 (58%)	3 (43%)	3 (100%)	1 (100%)	0 (NA%)	
V	117						0.2	23						0.7
0		52 (90%)	47 (96%)	7 (100%)	1 (100%)	1 (50%)			10 (83%)	7 (100%)	3 (100%)	1 (100%)	0 (NA%)	
1		6 (10%)	2 (4.1%)	0 (0%)	0 (0%)	1 (50%)			2 (17%)	0 (0%)	0 (0%)	0 (0%)	0 (N	

													A%)	
Pn	117							23						
0		58 (100%)	49 (100%)	7 (100%)	1 (100%)	2 (100%)			12 (100%)	7 (100%)	3 (100%)	1 (100%)	0 (NA%)	
R	117						0.064	23						0.4
0		51 (88%)	45 (92%)	6 (86%)	0 (0%)	1 (50%)			6 (50%)	7 (100%)	2 (67%)	1 (100%)	0 (NA%)	
1		5 (8.6%)	2 (4.1%)	1 (14%)	1 (100%)	0 (0%)			4 (33%)	0 (0%)	1 (33%)	0 (0%)	0 (NA%)	
Rx		2 (3.4%)	2 (4.1%)	0 (0%)	0 (0%)	1 (50%)			2 (17%)	0 (0%)	0 (0%)	0 (0%)	0 (NA%)	
futime_pfs	117	63 (33, 108)	55 (27, 110)	38 (29, 88)	22 (22, 22)	20 (4, 35)	0.3	23	30 (7, 79)	15 (6, 57)	22 (19, 52)	35 (35, 35)	NA (NA, NA)	>0.9
futime	117	64 (37, 110)	58 (39, 110)	40 (37, 88)	34 (34, 34)	25 (8, 42)	0.3	23	50 (22, 82)	57 (34, 71)	34 (27, 52)	42 (42, 42)	NA (NA, NA)	0.8
fustat	117	22 (38%)	26 (53%)	2 (29%)	1 (100%)	2 (100%)	0.11	23	10 (83%)	7 (100%)	2 (67%)	1 (100%)	0 (NA%)	0.3
fustat_pfs	117	25 (43%)	28 (57%)	3 (43%)	1 (100%)	2 (100%)	0.3	23	10 (83%)	7 (100%)	2 (67%)	1 (100%)	0 (NA%)	0.3

Her2_score								23						0.2
Zero									4 (33%)	3 (43%)	0 (0%)	0 (0%)	0 (NA%)	
Ultralow									7 (58%)	3 (43%)	2 (67%)	0 (0%)	0 (NA%)	
HER2 Score 1+									0 (0%)	1 (14%)	0 (0%)	0 (0%)	0 (NA%)	
HER2 Score 2+									0 (0%)	0 (0%)	1 (33%)	0 (0%)	0 (NA%)	
HER2 Score 3+									1 (8.3%)	0 (0%)	0 (0%)	1 (100%)	0 (NA%)	

[1]Data are presented as n (%) for categorical variables and median (Q1, Q3) for continuous variables. HER2/neu expression was analyzed separately in primary tumors and matched metastatic lesions. [2]Associations between categorical variables were assessed using Fisher's exact test; continuous or ordinal variables were compared using the Kruskal–Wallis rank-sum test. Percentages may not sum to 100% due to rounding.

Supplementary Table S6. Bivariate analysis of clinicopathological characteristics according to HER2/neu expression in primary tumors and metastasis in ovarian carcinoma.

Variable	HER2/neu Score - Primary							HER2/neu Score Primary - Metastasis						
	N	Zero N = 38[1]	Ultralow N = 12[1]	HER2 Score 1+ N = 1[1]	HER2 Score 2+ N = 1[1]	HER2 Score 3+ N = 1[1]	p-value[2]	N	Zero N = 14[1]	Ultralow N = 4[1]	HER2 Score 1+ N = 0[1]	HER2 Score 2+ N = 1[1]	HER2 Score 3+ N = 0[1]	p-value[2]
**HER2/neu Score - Metastasis**	19						**0.030**							
Zero		9 (69%)	5 (100%)	0 (NA%)	0 (0%)	0 (NA%)								
Ultralow		4 (31%)	0 (0%)	0 (NA%)	0 (0%)	0 (NA%)								
HER2 Score 1+		0 (0%)	0 (0%)	0 (NA%)	0 (0%)	0 (NA%)								
HER2 Score 2+		0 (0%)	0 (0%)	0 (NA%)	1 (100%)	0 (NA%)								
HER2 Score 3+		0 (0%)	0 (0%)	0 (NA%)	0 (0%)	0 (NA%)								
**Age**	52	64 (54, 71)	70 (63, 75)	49 (49, 49)	71 (71, 71)	52 (52, 52)	0.2	18	70 (59, 79)	73 (64, 75)	NA (NA, NA)	71 (71, 71)	NA (NA, NA)	>0.9
**FIGO.Stage**	52						0.6	18						0.2
1		2 (5.3%)	2 (18%)	0 (0%)	0 (0%)	0 (0%)			0 (0%)	0 (0%)	0 (NA%)	0 (0%)	0 (NA%)	
2		7 (18%)	0 (0%)	0 (0%)	0 (0%)	0 (0%)			0 (0%)	0 (0%)	0 (NA%)	0 (0%)	0 (NA%)	

Variable	HER2/neu Score - Primary							HER2/neu Score Primary - Metastasis						
	N	Zero N = 38[1]	Ultralow N = 12[1]	HER2 Score 1+ N = 1[1]	HER2 Score 2+ N = 1[1]	HER2 Score 3+ N = 1[1]	p-value[2]	N	Zero N = 14[1]	Ultralow N = 4[1]	HER2 Score 1+ N = 0[1]	HER2 Score 2+ N = 1[1]	HER2 Score 3+ N = 0[1]	p-value[2]
3		17 (45%)	6 (55%)	1 (100%)	0 (0%)	1 (100%)			9 (69%)	1 (25%)	0 (NA%)	0 (0%)	0 (NA%)	
4		12 (32%)	3 (27%)	0 (0%)	1 (100%)	0 (0%)			4 (31%)	3 (75%)	0 (NA%)	1 (100%)	0 (NA%)	
**pT**	52						0.7	18						>0.9
pT1		2 (5.3%)	2 (18%)	0 (0%)	0 (0%)	0 (0%)			0 (0%)	0 (0%)	0 (NA%)	0 (0%)	0 (NA%)	
pT2		7 (18%)	1 (9.1%)	0 (0%)	0 (0%)	0 (0%)			0 (0%)	0 (0%)	0 (NA%)	0 (0%)	0 (NA%)	
pT3		29 (76%)	8 (73%)	1 (100%)	1 (100%)	1 (100%)			13 (100%)	4 (100%)	0 (NA%)	1 (100%)	0 (NA%)	
**pN**	52						>0.9	18						0.7
pN0		12 (32%)	5 (45%)	1 (100%)	0 (0%)	0 (0%)			1 (7.7%)	1 (25%)	0 (NA%)	0 (0%)	0 (NA%)	
pN1		17 (45%)	4 (36%)	0 (0%)	1 (100%)	1 (100%)			8 (62%)	3 (75%)	0 (NA%)	1 (100%)	0 (NA%)	
pNx		9 (24%)	2 (18%)	0 (0%)	0 (0%)	0 (0%)			4 (31%)	0 (0%)	0 (NA%)	0 (0%)	0 (NA%)	
**M**	52						0.7	18						0.8

	HER2/neu Score - Primary							HER2/neu Score Primary - Metastasis						
**Variable**	**N**	**Zero** N = 38[1]	**Ultralow** N = 12[1]	**HER2 Score 1+** N = 1[1]	**HER2 Score 2+** N = 1[1]	**HER2 Score 3+** N = 1[1]	**p-value**[2]	**N**	**Zero** N = 14[1]	**Ultralow** N = 4[1]	**HER2 Score 1+** N = 0[1]	**HER2 Score 2+** N = 1[1]	**HER2 Score 3+** N = 0[1]	**p-value**[2]
pM0		26 (68%)	8 (73%)	1 (100%)	0 (0%)	1 (100%)			8 (62%)	2 (50%)	0 (NA%)	0 (0%)	0 (NA%)	
pM1		12 (32%)	3 (27%)	0 (0%)	1 (100%)	0 (0%)			5 (38%)	2 (50%)	0 (NA%)	1 (100%)	0 (NA%)	
**L**	52						>0.9	18						0.2
L0		20 (53%)	5 (45%)	1 (100%)	1 (100%)	1 (100%)			2 (15%)	1 (25%)	0 (NA%)	1 (100%)	0 (NA%)	
L1		18 (47%)	6 (55%)	0 (0%)	0 (0%)	0 (0%)			11 (85%)	3 (75%)	0 (NA%)	0 (0%)	0 (NA%)	
**V**	52						>0.9	18						>0.9
V0		35 (92%)	10 (91%)	1 (100%)	1 (100%)	1 (100%)			11 (85%)	4 (100%)	0 (NA%)	1 (100%)	0 (NA%)	
V1		3 (7.9%)	1 (9.1%)	0 (0%)	0 (0%)	0 (0%)			2 (15%)	0 (0%)	0 (NA%)	0 (0%)	0 (NA%)	
**Pn**	52						>0.9	18						>0.9
Pn0		37 (97%)	11 (100%)	1 (100%)	1 (100%)	1 (100%)			12 (92%)	4 (100%)	0 (NA%)	1 (100%)	0 (NA%)	
Pn1		1 (2.6%)	0 (0%)	0 (0%)	0 (0%)	0 (0%)			1 (7.7%)	0 (0%)	0 (NA%)	0 (0%)	0 (NA%)	
**R**	52						0.4	18						**0.012**

Variable	HER2/neu Score - Primary							HER2/neu Score Primary - Metastasis						
	N	Zero N = 38[1]	Ultralow N = 12[1]	HER2 Score 1+ N = 1[1]	HER2 Score 2+ N = 1[1]	HER2 Score 3+ N = 1[1]	p-value[2]	N	Zero N = 14[1]	Ultralow N = 4[1]	HER2 Score 1+ N = 0[1]	HER2 Score 2+ N = 1[1]	HER2 Score 3+ N = 0[1]	p-value[2]
R0		32 (84%)	9 (82%)	0 (0%)	1 (100%)	1 (100%)			13 (100%)	1 (25%)	0 (NA%)	1 (100%)	0 (NA%)	
R1		4 (11%)	2 (18%)	1 (100%)	0 (0%)	0 (0%)			0 (0%)	2 (50%)	0 (NA%)	0 (0%)	0 (NA%)	
Rx		2 (5.3%)	0 (0%)	0 (0%)	0 (0%)	0 (0%)			0 (0%)	1 (25%)	0 (NA%)	0 (0%)	0 (NA%)	
**Progression-Free Survival in Months**	52	13 (8, 34)	22 (10, 38)	2 (2, 2)	18 (18, 18)	21 (21, 21)	0.4	18	12 (8, 32)	7 (4, 9)	NA (NA, NA)	18 (18, 18)	NA (NA, NA)	0.2
**Survival in Months**	52	34 (20, 61)	29 (17, 63)	11 (11, 11)	102 (102, 102)	48 (48, 48)	0.4	18	21 (15, 42)	13 (6, 24)	NA (NA, NA)	102 (102, 102)	NA (NA, NA)	0.10
**Deceased**	52	36 (95%)	8 (73%)	1 (100%)	1 (100%)	1 (100%)	0.3	18						
1									13 (100%)	4 (100%)	0 (NA%)	1 (100%)	0 (NA%)	
**Progression**	52	37 (97%)	9 (82%)	1 (100%)	1 (100%)	1 (100%)	0.3	18						
1									13 (100%)	4 (100%)	0 (NA%)	1 (100%)	0 (NA%)	

Variable	HER2/neu Score - Primary							HER2/neu Score Primary - Metastasis						
	**N**	**Zero** N = 38[1]	**Ultralow** N = 12[1]	**HER2 Score 1+** N = 1[1]	**HER2 Score 2+** N = 1[1]	**HER2 Score 3+** N = 1[1]	**p-value**[2]	**N**	**Zero** N = 14[1]	**Ultralow** N = 4[1]	**HER2 Score 1+** N = 0[1]	**HER2 Score 2+** N = 1[1]	**HER2 Score 3+** N = 0[1]	**p-value**[2]
**HER2/neu Score**								19						**0.030**
Zero									9 (64%)	4 (100%)	0 (NA%)	0 (0%)	0 (NA%)	
Ultralow									5 (36%)	0 (0%)	0 (NA%)	0 (0%)	0 (NA%)	
HER2 Score 1+									0 (0%)	0 (0%)	0 (NA%)	0 (0%)	0 (NA%)	
HER2 Score 2+									0 (0%)	0 (0%)	0 (NA%)	1 (100%)	0 (NA%)	
HER2 Score 3+									0 (0%)	0 (0%)	0 (NA%)	0 (0%)	0 (NA%)	

[1]Data are presented as n (%) for categorical variables and median (Q1, Q3) for continuous variables. HER2/neu expression was analyzed separately in primary tumors and matched metastatic lesions. [2]Associations between categorical variables were assessed using Fisher's exact test; continuous or ordinal variables were compared using the Kruskal–Wallis rank-sum test. Percentages may not sum to 100% due to rounding.

Supplementary Table S7. Changes in HER2/neu scores between primary and metastatic tumor tissue for EC

**Primary tumor/ Metastasis**	**HER2/neu - zero, n(%)**	**HER2/neu - ultralow, n(%)**	**HER2/neu 1+, n(%)**	**HER2/neu 2+, n(%)**	**HER2/neu 3+, n(%)**
**HER2/neu - zero, n(%)**	4 (17.4%)	7 (30.4%)	0 (0.0%)	0 (0.0%)	1 (4.3%)
**HER2/neu - ultralow, n(%)**	3 (13%)	3 (13%)	1 (4.3%)	0 (0.0%)	0 (0.0%)
**HER2/neu 1+, n(%)**	0 (0.0%)	2 (8.7%)	0 (0.0%)	1 (4.3%)	1 (4.3%)
**HER2/neu 2+, n(%)**	0 (0.0%)	0 (0.0%)	0 (0.0%)	0 (0.0%)	0 (0.0%)
**HER2/neu 3+, n(%)**	0 (0.0%)	0 (0.0%)	0 (0.0%)	0 (0.0%)	0 (0.0%)

Rows indicate HER2/neu scores in metastatic lesions, and columns indicate HER2/neu scores in matched primary tumors. Data are presented as n (%), with percentages referring to all matched primary tumor–metastasis pairs in endometrial carcinoma (n = 23)

Supplementary Table S8. Changes in HER2/neu scores between primary and metastatic tumor tissue for OC

Primary tumor/ Metastasis	HER2/neu - zero, n(%)	HER2/neu - ultralow, n(%)	HER2/neu 1+, n(%)	HER2/neu 2+, n(%)	HER2/neu 3+, n(%)
**HER2/neu - zero, n(%)**	9 (47.4%)	5 (26.3%)	0 (0.0%)	0 (0.0%)	0 (0.0%)
**HER2/neu - ultralow, n(%)**	4 (21.1%)	0 (0.0%)	0 (0.0%)	0 (0.0%)	0 (0.0%)
**HER2/neu 1+, n(%)**	0 (0.0%)	0 (0.0%)	0 (0.0%)	0 (0.0%)	0 (0.0%)
**HER2/neu 2+, n(%)**	0 (0.0%)	0 (0.0%)	0 (0.0%)	1 (5.3%)	0 (0.0%)
**HER2/neu 3+, n(%)**	0 (0.0%)	0 (0.0%)	0 (0.0%)	0 (0.0%)	0 (0.0%)

Rows indicate HER2/neu scores in metastatic lesions, and columns indicate HER2/neu scores in matched primary tumors. Data are presented as n (%), with percentages referring to all matched primary tumor–metastasis pairs in HGSOC (n = 19).
